# Linker, loading, and reaction scale influence automated glycan assembly

**DOI:** 10.3762/bjoc.19.77

**Published:** 2023-07-06

**Authors:** Marlene C S Dal Colle, Manuel G Ricardo, Nives Hribernik, José Danglad-Flores, Peter H Seeberger, Martina Delbianco

**Affiliations:** 1 Department of Biomolecular Systems, Max Planck Institute of Colloids and Interfaces, Am Mühlenberg 1, 14476 Potsdam, Germany; 2 Department of Chemistry and Biochemistry, Freie Universität Berlin, Arnimallee 22, 14195 Berlin, Germany

**Keywords:** automated glycan assembly, photocleavable linker, polysaccharides, solid-phase synthesis

## Abstract

Automated glycan assembly (AGA) affords collections of well-defined glycans in a short amount of time. We systematically analyzed how parameters connected to the solid support affect the AGA outcome for three different glycan sequences. We showed that, while loading and reaction scale did not significantly influence the AGA outcome, the chemical nature of the linker dramatically altered the isolated yields. We identified that the major determinants of AGA yields are cleavage from the solid support and post-AGA purification steps.

## Introduction

**Figure 1:**
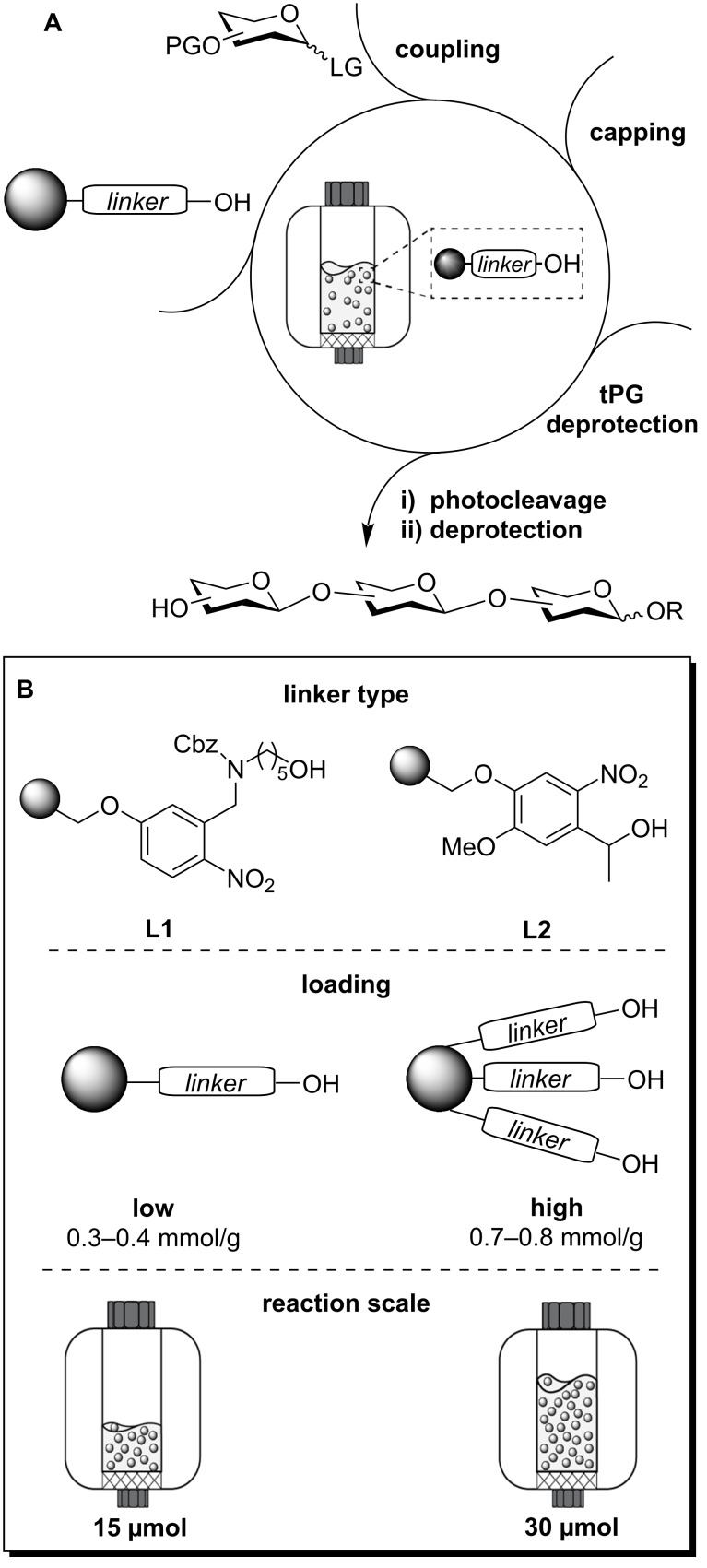
Schematic representation of the AGA process (A). Variables that can affect the AGA outcome investigated in this study (B).

Automated glycan assembly (AGA) is a solid-phase method that enables the rapid synthesis of complex oligo- and polysaccharides from protected monosaccharide building blocks (BBs) [1–2]. Iterative cycles of glycosylation, capping, and selective deprotection afford the support-bound glycan with a programmable sequence (Figure 1A). The protected glycan is then cleaved from the solid support and subjected to post-AGA deprotection steps to reveal the target glycan. AGA is mostly performed on cross-linked polystyrene resins equipped with photocleavable linkers [3], offering orthogonality to all the synthetic steps of the assembly, while selectively releasing the glycan at the end of the synthesis.

In recent years, the implementation of new synthetic strategies [4–7] as well as technological improvements [8–9] permitted access to highly complex carbohydrates [10]. Still, variations in yields are not always ascribable to the AGA process [11–16]. Dissimilar structures are assembled in high purity as indicated by HPLC analysis of the crude products, but isolated in relatively low yields. The optimization procedures are focused on glycan elongation (i.e., glycosylation and deprotection steps), whereas less attention is given to variables associated with the solid support [17]. In contrast, substantial knowledge exists on how loading [18], reaction scale [19], and linkers [20–21] affect the overall yield of solid phase peptide synthesis (SPPS). In the past decades, several supports and linkers have been developed and commercialized for SPPS, enabling a wide range of applications. Solid supports are available with different linker loadings, with low loading (0.1–0.2 mmol/g) being beneficial to avoid aggregation of long peptide sequences, and high loadings (0.4–0.5 mmol/g) advantageous for more efficient syntheses [21].

Herein, we systematically investigate how variations in linker type, resin loading, and reaction scale influence the productivity of AGA.

## Results and Discussion

We selected three glycan sequences as models to analyze the effect of different parameters on the AGA outcome. Each sequence was prepared on four batches of Merrifield resin functionalized with two photolabile linkers (**L1** [22] vs **L2** [3]), at two linker loadings (low vs high) (Figure 1B). Each AGA experiment was performed at two different reaction scales (15 vs 30 µmol). All AGA runs were performed adjusting the resin amount to the desired reaction scale, while keeping the concentration of all other reagents constant (Figure 1B).

The photolabile linkers **L1** [22] and **L2** [3] are based on the *o-*nitrobenzyl scaffold [23–24] and expose a hydroxy group that serves as glycosyl acceptor in the first AGA cycle (Figure 1B). While **L1** displays a flexible aliphatic chain terminating with a primary alcohol, **L2** carries a secondary benzylic alcohol. Upon irradiation with UV light (λ = 360 nm), **L1** releases the glycan equipped with an aminoalkyl spacer at the reducing end, whereas **L2** affords the free reducing sugar (α/β mixture). Previous data suggested that UV cleavage of **L1** and **L2** was equally efficient, permitting the isolation of a tetramannoside in around 60% yield [3]. We wondered whether different glycan sequences were more sensitive to the linker structure. Less reactive donors might highlight differences in the linker nucleophilicity [25]. The aggregation of the growing glycan chains is conceivable to be connected to linker flexibility [18]. The efficiency of UV cleavage is probably influenced by glycan structure, solubility, and aggregation tendency [26]. Lastly, purification of the protected glycan upon cleavage could be affected by the presence or absence of a linker.

**L1** or **L2** were conjugated to Merrifield resins with initial loadings of 0.5 mmol/g and 1.0 mmol/g to yield supports with low (0.3–0.4 mmol/g) or high (0.7–0.8 mmol/g) loadings (see Supporting Information File 1, section 2.3, module A). The latter allows for a larger synthesis scale, but steric hindrance and chain–chain interactions could negatively influence the AGA outcome, as observed for some peptide sequences [18]. Moreover, high-loading supports might result in inefficient UV cleavage due to quenching. These four supports were studied in AGA experiments performed at 15 and 30 µmol reaction scales. While AGA is commonly performed at a 15 µmol reaction scale, a larger reaction scale is attractive to produce more material in a single AGA run, but might suffer from insufficient mixing [27–28], causing slower kinetics [29], temperature gradients [30], and precipitation [31].

**Figure 2:**
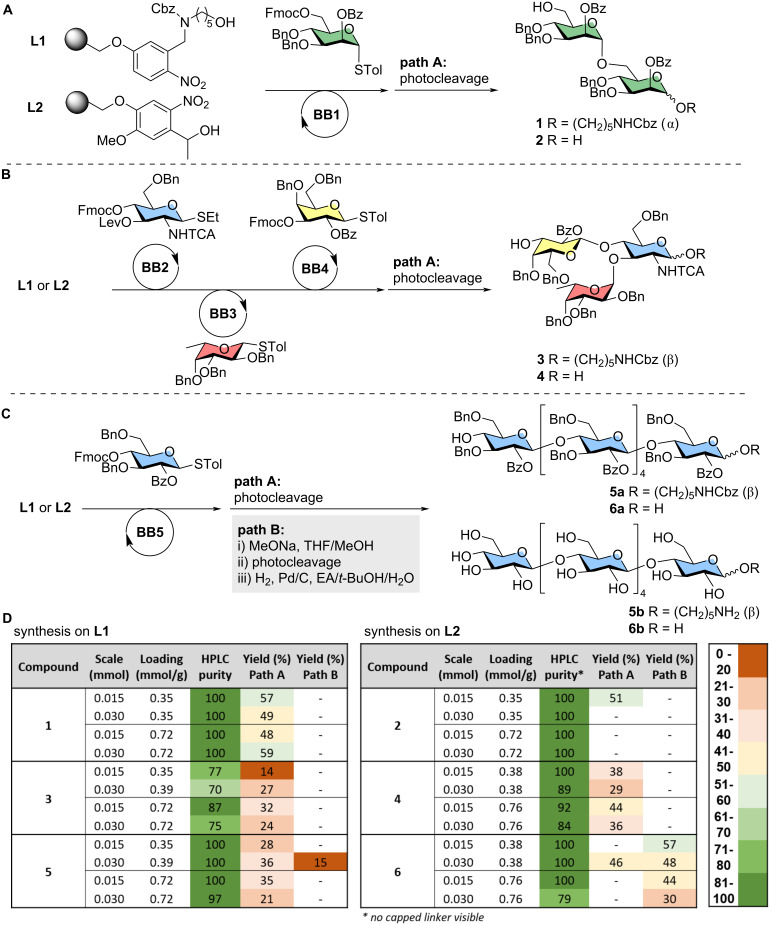
AGA of model glycan sequences analyzed in this study: α-1,6-linked dimannosides **1**, **2** (A), branched trisaccharides **3**, **4** (B), and linear α-1,4-linked hexaglucosides **5**, **6** (C). Tables summarizing the results obtained for the AGA experiments performed in different conditions (D). The HPLC purity is estimated based on the ELSD profile. This value should be used to compare results within each series of experiments (i.e. same glycan sequence).

We set off to study the effect of these parameters on the AGA of three different glycan sequences (Figure 2). In an increasing order of complexity, we prepared α-1,6-linked dimannosides (**1**,**2**) [32], branched trisaccharides (**3**,**4**) [12], and linear α-1,4-linked hexaglucosides (**5**,**6**) [15,33]. Each synthesis was performed with 6.5 equivalents of BB per glycosylation cycle using previously reported AGA conditions (see Supporting Information File 1, section 2.3, module C). The outcome of each AGA experiment was analyzed in terms of: i) HPLC purity based on the chromatogram of the crude sample after AGA and UV cleavage, ii) isolated yield of the target compound after photocleavage and HPLC purification (path A).

**Figure 3:**
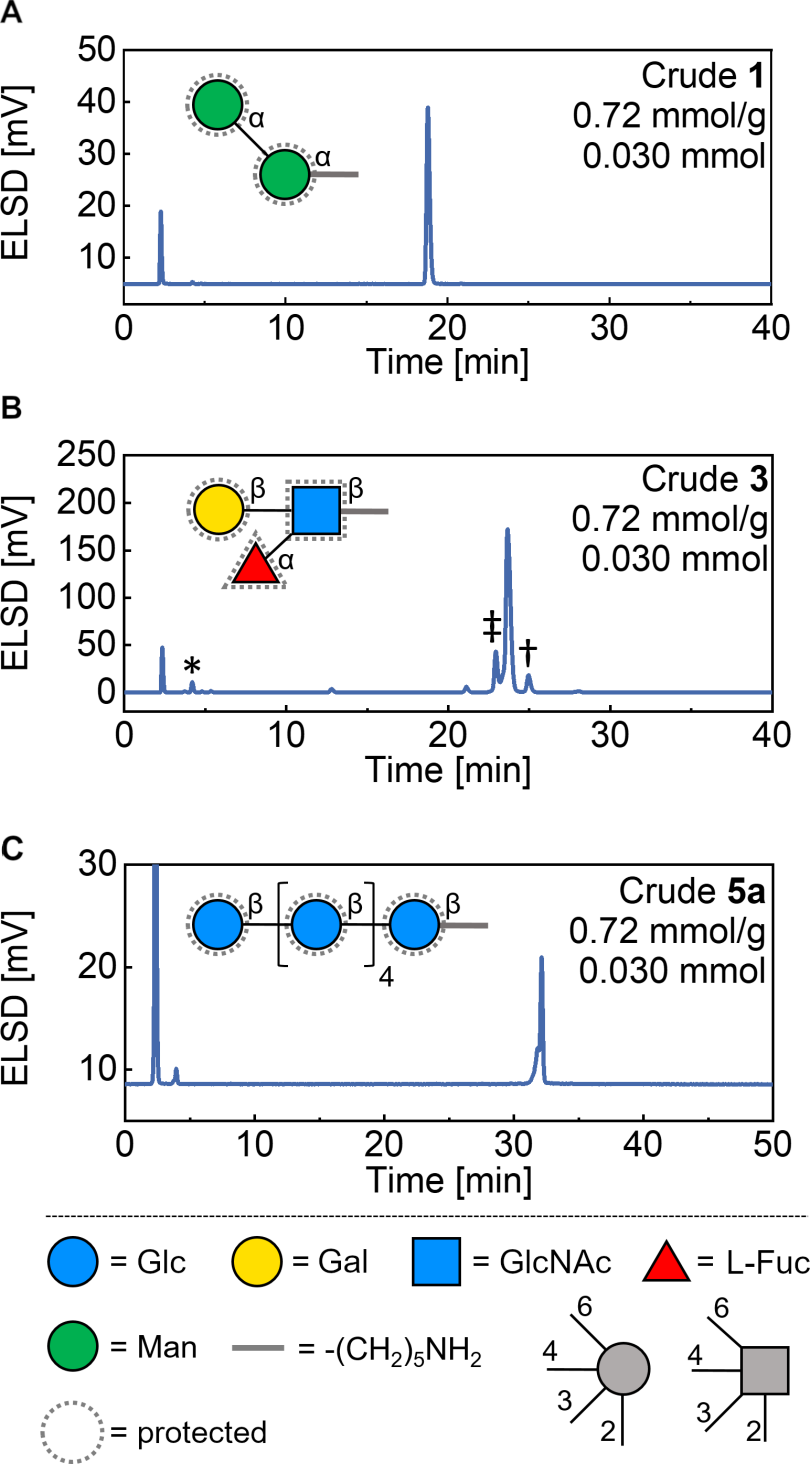
Representative HPLC traces for the crude compounds **1** (A), **3** (B), and **5a** (C) after cleavage from the solid support. HPLC conditions are reported in Supporting Information File 1 (Figures S2, S4, S8, A). In B, MS analysis showed the presence of capped linker (*), capped dimer (†), and Lev-containing dimer (‡). The monosaccharides are represented following the symbol nomenclature for glycans (SNFG).

The syntheses of the α-1,6-linked dimannosides **1** and **2** (Figure 2A) were successful on all resins tested, affording the desired product in complete purity regardless of linker type, loading or reaction scale (Figure 3A, and Figures S2 and S3 in Supporting Information File 1). Isolated yields of 49–59% were obtained in all experiments (Figure 2D), after cleavage of the photolabile unit.

The syntheses of the branched trisaccharides **3** and **4** (Figure 2B, and Figures S4 and S6 in Supporting Information File 1) were less efficient. Even though the target compound was the major product in all experiments, deletion sequences were observed in the chromatograms of the crudes (Figure 3B). MS analysis showed the presence of capped linker (*), capped dimer (†), and Lev-containing dimer (‡) (see Figures S5 and S7 in Supporting Information File 1). No significant variations were noticed within each series of experiments, with slightly better purities obtained for AGA performed on **L2** (to note: for experiments on **L2** no capped linker was detectable by HPLC; see Supporting Information File 1). Isolated yields were relatively low for all experiments (14–32% on **L1** and 29–44% on **L2**, Figure 2D). These values are quite low even considering the presence of deletion sequences, suggesting that cleavage and purification are more challenging for these structures. Overall, a slightly better performance of **L2** resulting in higher purities and better yields was noticed.

HPLC analysis showed that the β-1,4-hexaglucosides **5a** and **6a** were produced in excellent purity in all experiments (Figures 2D, 3C, and Figures S8 and S9 in Supporting Information File 1). For these compounds, we explored two different post-AGA procedures: the standard path A based on photocleavage and HPLC purification, and path B involving on resin methanolysis of the ester groups, photocleavage, hydrogenolysis of the remaining PGs, and purification (Figure 2C). The latter is commonly employed for compounds synthesized on **L2** because of the poor stability of free-reducing glycans in basic conditions needed for the methanolysis step [33].

The isolated yields of the fully protected compound **5a** synthesized on **L1** were significantly lower than expected (21–36%, Figure 2D, path A), with little variation within the series. Isolated yields for the linker-free compound **6a** prepared on **L2** were around 10% higher (46%). The absence of deletion sequences in the HPLC of the crude compounds indicated that cleavage and/or purification are the major bottlenecks of these syntheses.

Higher yields (30–57%) were obtained for compound **6b**, isolated after the post-AGA procedure path B (Figure 2D). This is surprising since the path B procedure involved additional deprotection steps. Therefore, we wondered whether methanoloysis on resin could improve photocleavage efficiency. However, when we tested the same procedure on **L1**, target compound **5b** was isolated in only 15% yield. These results strongly suggest that the two linkers perform differently depending on the glycan sequences.

## Conclusion

Taken together, the results showed minimal variation within each series of experiments, indicating that loading and reaction scale are not significantly affecting AGA of those sequences within the range of conditions explored here. This is a promising observation from the perspective of scaling up AGA. No differences were observed for the AGA of simple disaccharides **1**,**2** performed on **L1** and **L2** with an apparent maximal yield of around 60%, in agreement with previous reports [3]. In contrast, other sequences constructed on **L2** were isolated in slightly better yields. This result could be connected to more efficient cleavage of **L2** in the presence of complex glycan sequences, easier purification of linker-free compounds, or a combination of both.

Our systematic study identified that the major determinants of AGA yields are cleavage from the solid support and purification steps. These two aspects are strongly connected to the glycan structure, with minimal variations such as presence or absence of a linker playing an important role in the post-AGA process. In some cases, performing post-AGA manipulations on resin dramatically improved the overall yield of the process. Future efforts need to focus on the development of new linkers, more efficient cleaving procedures [34], and the implementation of post-AGA manipulation steps on resin.

## Supporting Information

File 1 Experimental procedures and characterization data.
