# Supplementary material for: Linker, loading, and reaction scale influence automated glycan assembly
Source: Beilstein J Org Chem. 2023 Jul 6;19:1015–20. doi: 10.3762/bjoc.19.77 (PMC10334207; doi:10.3762/bjoc.19.77)
Supplement: File 1 — Experimental procedures and characterization data. [file Beilstein_J_Org_Chem-19-1015-s001.pdf]

BEILSTEIN JOURNAL OF ORGANIC CHEMISTRY

Supporting Information

for

Linker, loading, and reaction scale influence automated glycan assembly

Marlene C. S. Dal Colle, Manuel G. Ricardo, Nives Hribernik, José Danglad-Flores, Peter H. Seeberger and Martina Delbianco

Beilstein J. Org. Chem. **2023,** *19,* 1015–1020. doi:10.3762/bjoc.19.77

Experimental procedures and characterization data

License and Terms: This is a supporting information file under the terms of the Creative Commons Attribution License (https://creativecommons.org/licenses/by/4.0). Please note that the reuse, redistribution and reproduction in particular requires that the author(s) and source are credited and that individual graphics may be subject to special legal provisions.
The license is subject to the *Beilstein Journal of Organic Chemistry* terms and conditions: (https://www.beilstein-journals.org/bjoc/terms)

Table of contents

1 **General materials and methods** ... S2

2 **Automated glycan assembly** ... S3

2.1 General materials and methods ... S3

2.2 Preparation of stock solutions ... S3

2.3 Modules for automated synthesis ... S3

2.4 Post-AGA manipulations ... S6

3 **Oligosaccharide synthesis** ... S8

3.1 Synthesis of **1** ... S9

3.2 Synthesis of **2** ... S13

3.3 Synthesis of **3** ... S17

3.4 Synthesis of **4** ... S21

3.5 Synthesis of **5** ... S25

3.6 Synthesis of **6** ... S31

4 **References** ... S37

1 General materials and methods

All chemicals used were reagent grade and used as supplied unless otherwise noted. The automated syntheses were performed on a home-built synthesizer developed at the Max Planck Institute of Colloids and Interfaces.[1] Analysis and purification by normal phase HPLC was performed by using an Agilent 1200 series. Products were lyophilized using a Christ Alpha 2-4 LD plus freeze dryer. ^{1}H, ^{13}C and HSQC NMR spectra were recorded on a Varian 400-MR (400 MHz), or Varian 600-NMR (600 MHz) spectrometer. Spectra were recorded in $CDCl_3$ using the solvent residual peak chemical shift as the internal standard in ^{1}H and ^{13}C NMR ($CDCl_3$: 7.26 ppm ^{1}H, 77.0 ppm ^{13}C) or in D_2O using the solvent as the internal standard in ^{1}H NMR (D_2O: 4.79 ppm ^{1}H). Weak intensity ^{13}C resonances were derived from the respective HSQC crosspeaks. ^{1}H NMR integrals of the resonances corresponding to residues at the reducing end are reported as non-integer numbers and the sum of the integrals of α and β anomers, H-1 α and H-1 β respectively, is set to 1. High resolution mass spectra were obtained using a 6210 ESI-TOF mass spectrometer (Agilent) and a MALDI-TOF autoflex™ (Bruker). MALDI and ESI mass spectra were run on IonSpec Ultima instruments.

2 Automated glycan assembly

2.1 General materials and methods

The automated syntheses were performed on a home-built synthesizer developed at the Max Planck Institute of Colloids and Interfaces. All solvents used were HPLC-grade. The solvents used for the building blocks, activator, TMSOTf and capping solutions were taken from an anhydrous solvent system (J.C. Meyer). The building blocks were co-evaporated three times with toluene and dried on high vacuum before use. Oven-heated, argon-flushed flasks were used to prepare all moisture-sensitive solutions. Activator, capping, deprotection, acidic wash and building block solutions were freshly prepared and kept under argon during the automation run. All procedures of the automated glycan assembly were performed according to previously published protocols.[2] All yields of products obtained by AGA were calculated on the basis of resin loading. Resin loading was determined following previously established procedures.[3]

2.2 Preparation of stock solutions

- **Building block solution**: 0.1 mmol of building block (see Module C) was dissolved in DCM (1 mL) for the syntheses performed on a 0.015 mmol scale. Building block (0.2 mmol depending on the BB, see Module C) was dissolved in DCM (2 mL) for the syntheses performed on a 0.03 mmol scale.

- **NIS/TfOH activator solution**: 1.35 g (6.0 mmol) of recrystallized NIS was dissolved in 40 mL of a 2:1 (v/v) mixture of anhydrous DCM and anhydrous dioxane. Then, triflic acid (55 µL, 0.6 mmol) was added. The solution was kept at 0 °C (ice bath) for the duration of the automation run.

- **Fmoc deprotection solution**: a solution of 20% (v/v) piperidine in DMF was prepared.

- **Lev deprotection solution**: hydrazine acetate (550 mg, 5.97 mmol) was dissolved in pyridine/AcOH/H_2O (40 mL, v/v, 32:8:2) and sonicated for 10 min.

- **TMSOTf solution**: TMSOTf (0.45 mL, 2.49 mmol) was added to DCM (40 mL).

- **Capping solution**: a solution of 10% (v/v) acetic anhydride and 2% (v/v) methanesulfonic acid in DCM was prepared.

2.3 Modules for automated synthesis

Module A: resin preparation

All automated syntheses were performed on either 0.015 or 0.03 mmol scales. Resin (**L1** or **L2**) is placed in the reaction vessel and swollen in DCM for 20 min at rt prior to the synthesis. During this time, all reagent lines needed for the synthesis are washed and primed. After the swelling, the resin is washed with DMF, THF, and DCM (three times each with 2 mL for 25 s).

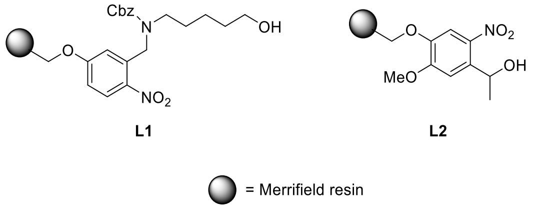

	L1				L2				
**Provider loading (mmol/g)**	0.5		1		0.5		1	1	
**Final loading (mmol/g)[a]**	0.35, 0.39[b]		0.72		0.38		0.4	0.76, 0.8[b]	
**Scale (mmol)**	0.015	0.03	0.015	0.03	0.015	0.03	0.015	0.015	0.03
**Resin amount (mg)**	43, 39[b]	86, 77[b]	21	42	40	79	38	20, 19[b]	40, 38[b]

[a] Loading after functionalization of Merrifield resin with linker **L1** or **L2**.
[b] The two numbers correspond to different batches of resin.

Module B: Acidic wash with TMSOTf solution (20 min)
The resin is swollen in 2 mL DCM and the temperature of the reaction vessel adjusted to −20 °C. Upon reaching the low temperature, TMSOTf solution (1 mL) is added dropwise to the reaction vessel. After bubbling for 3 min, the acidic solution is drained and the resin washed with 2 mL DCM for 25 s.

Action	Cycles	Solution	Amount	T (°C)	Incubation time
**Cooling**	–	–	–	−20	(15 min)*
**Deliver**	1	DCM	2 mL	−20	–
**Deliver**	1	TMSOTf solution	1 mL	−20	3 min
**Wash**	1	DCM	2 mL	−20	25 s

*Time required to reach the desired temperature.

Module C: Thioglycoside glycosylation (35 –55 min)
The building block solution (0.1 mmol of BB in 1 mL of DCM per glycosylation) is delivered to the reaction vessel. After the set temperature is reached, the reaction is started by dropwise addition of the NIS/TfOH activator solution (1.0 mL, excess). The glycosylation conditions (T_1, T_2, t_1, and t_2) are building block dependent and are reported for each synthesis. After completion of the reaction, the solution is drained and the resin is washed with DCM, DCM/dioxane 1:2 (3 mL for 20 s) and DCM (two times, each with 2 mL for 25 s). The temperature of the reaction vessel is increased to 25 °C for the next module.

Action	Cycles	Solution	Amount	T (°C)	Incubation time
**Cooling**	–	–	–	T_1	–
**Deliver**	1	BB solution	1 mL*	T_1	–
**Deliver**	1	NIS/TfOH activator solution	1 mL*	T_1	–
**Reaction time (BB dependent)**	1	–	–	T_1 to T_2	t_1 t_2

**Wash**	1	DCM	2 mL	T_2	5 s
**Wash**	1	DCM/dioxane 1:2	2 mL	T_2	20 s
**Heating**	–	–	–	25	–
**Wash**	2	DCM	2 mL	> 0	25 s

The AGA glycosylation conditions employed for thioglycoside BBs were previously reported.[4,5]
*For the syntheses performed on 0.03 mmol scale, 2 mL of BB and 2 mL of activator solutions were delivered to the reaction vessel.

BB	Equiv	t_1 (min)	T_1 (°C)	i$_2$ (min)	T_2 (°C)
**1**	6.5	5	−20	20	0
**2**	6.5	5	−20	40	0
**3**	6.5	5	−20	20	0
**4**	6.5	5	−40	20	−20
**5**	6.5	5	−20	20	0

Module D: Capping (30 min)
The resin is washed with DMF (two times with 2 mL for 25 s) and the temperature of the reaction vessel adjusted to 25 °C. A pyridine solution (2 mL, 10% (v/v) in DMF) is delivered into the reaction vessel. After 1 min, the reaction solution is drained and the resin washed with DCM (three times with 3 mL for 25 s). Capping solution (4 mL) is delivered into the reaction vessel. After 20 min, the reaction solution is drained and the resin washed with DCM (three times with 3 mL for 25 s).

Action	Cycles	Solution	Amount	T (°C)	Incubation time
**Heating**	–	–	–	25	(5 min)*
**Wash**	2	DMF	2 mL	25	25 s
**Deliver**	1	10% pyridine in DMF	2 mL	25	1 min
**Wash**	3	DCM	2 mL	25	25 s
**Deliver**	1	capping solution	4 mL	25	20 min
**Wash**	3	DCM	2 mL	25	25 s

*Time required to reach the desired temperature.

Module E1: Fmoc deprotection (9 min)
The resin is washed with DMF (three times with 2 mL for 25 s) and the temperature of the reaction vessel adjusted to 25 °C. Fmoc deprotection solution (2 mL) is delivered to the reaction vessel and kept under Ar bubbling. After 5 min, the reaction solution is drained and the resin washed with DMF (three times with 3 mL for 25 s) and DCM (five times each with 2 mL for 25 s). The temperature of the reaction vessel is decreased to −20 °C for the next module.

Action	Cycles	Solution	Amount	T (°C)	Incubation time
**Wash**	3	DMF	2 mL	25	25 s
**Deliver**	1	Fmoc depr. solution	2 mL	25	5 min
**Wash**	1	DMF	2 mL		
**Cooling**	–	–	–	−20	–
**Wash**	3	DMF	2 mL	< 25	25 s
**Wash**	5	DCM	2 mL	< 25	25 s

Module E2: Lev deprotection (65 min)

The resin was washed with DCM (three times with 2 mL for 25 s). DCM (1.3 mL) was delivered to the reaction vessel and the temperature of the reaction vessel was adjusted to 25 °C. Two mL of Lev deprotection solution were delivered to the reaction vessel that was kept under pulsed Ar bubbling for 30 min. This procedure was repeated twice. The reaction solution was drained and the resin washed with DMF (three times with 3 mL for 25 s) and DCM (five times each with 2 mL for 25 s).

Action	Cycles	Solution	Amount	T (°C)	Incubation time
Wash	3	DMF	2 mL	25	25 s
Deliver	2	Lev depr. solution	2 mL	25	30 min
Wash	1	DMF	2 mL		
Cooling	–	–	–	−20	–
Wash	3	DMF	2 mL	< 25	25 s
Wash	5	DCM	2 mL	< 25	25 s

2.4 Post-AGA manipulations

Module F: On-resin methanolysis
The resin is suspended in THF (4 mL). MeONa in MeOH (0.5 M, 0.4 mL) is added and the suspension is gently shaken at room temperature. After micro-cleavage (see *Module G2*) indicates the complete removal of benzoyl groups, the resin is repeatedly washed with MeOH (3 × 2 mL) and DCM (3 × 2 mL). For the syntheses performed on 0.03 mmol scale, the amounts are adjusted to 8 mL of THF and 0.8 mL of MeONa in MeOH.

Module G1: Cleavage from solid support
The oligosaccharides are cleaved from the solid support using a continuous-flow photoreactor as described previously.[6]

Module G2: Micro-cleavage from solid support
Trace amount of resin (around 20 beads) is dispersed in DCM (0.1 mL) and irradiated with a UV lamp (6 W, 356 nm) for 10 minutes. ACN (10 μL) is then added to the resin and the resulting solution analyzed by MALDI.

Module H: Hydrogenolysis at ambient pressure

The crude compound obtained from *Module G1* is dissolved in 2 mL of EtOAc/*t*-BuOH/H_2O 2:1:1. 100% by weight Pd/C (10 wt %) is added to the flask while stirring, the reaction purged for 5 min with a N_2 balloon, and equipped with a H_2 balloon. The reaction progress is monitored to avoid undesired side products formation (i.e. degradation of reducing end).[7] Upon completion, the reaction is filtered (PTFE 0.45 μm 25 mm syringe filter, Fisher scientific) and washed with EtOAc, H_2O, and ACN (4 mL each). The filtrates are concentrated in vacuo.

Module I: Purification

The final compounds are analyzed using analytical normal or reverse phase HPLC (Agilent 1200 Series, Methods A1 and B). The purification of the crudes is conducted using normal phase HPLC (Agilent 1200 Series, Method A2) or manual C_{18} silica column (Method C).

- **Method A1**: (YMC-Diol-300 column, 150 × 4.6 mm), flow rate of 1.0 mL/min with Hex and EtOAc as eluents [isocratic 20% EtOAc (5 min), linear gradient to 60% EtOAc (45 min), linear gradient to 100% EtOAc (5 min)].

- **Method A2 (Prep)**: (YMC-Diol-300 column, 150 × 20 mm), flow rate of 15 mL/min with Hex and EtOAc as eluents [isocratic 20% EtOAc (5 min), linear gradient to 60% EtOAc (45 min), linear gradient to 100% EtOAc (5 min)].

- **Method B**: (Hypercarb column, 150 × 4.6 mm, 3 μm) flow rate of 0.7 mL/min with H_2O (0.1% formic acid) as eluents [isocratic (5 min), linear gradient to 30% ACN (30 min), linear gradient to 100% ACN (5 min)].

- **Method C**: (Manual reverse phase C_{18} silica gel column chromatography, 80 × 15 mm): H_2O (0.1% formic acid, 10 mL), 3% MeOH (10 mL), 6% MeOH (10 mL), 9% MeOH (10 mL), 12% MeOH (10 mL), 15% MeOH (10 mL).

Following final purification, all deprotected products are lyophilized on a Christ Alpha 2-4 LD plus freeze dryer prior to characterization.

3 Oligosaccharide synthesis

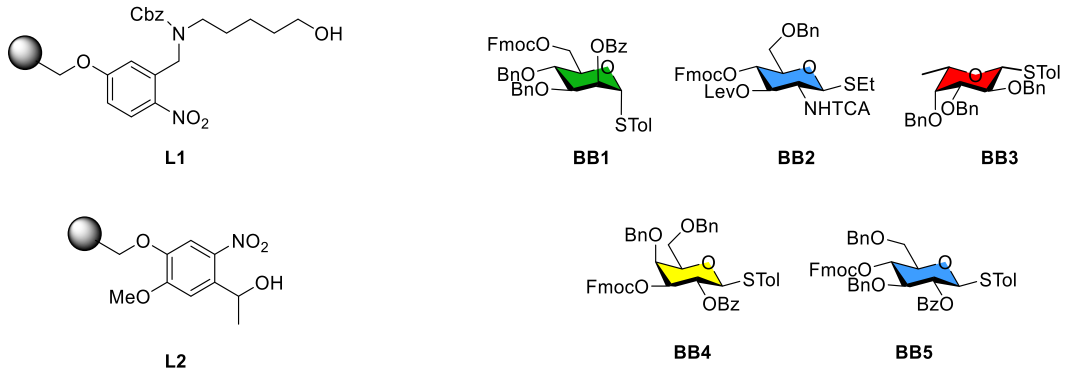

Figure S1 BBs and solid supports used for the AGA of compounds **1**, **2**, **3**, **4**, **5a**, and **6a**.

BB1, **BB2**, **BB3**, **BB4**, and **BB5** were purchased from GlycoUniverse (Germany). Merrifield resin equipped with photocleavable linkers **L1** and **L2** was prepared according to previously established procedures.[8] Compounds **1**,[9] **5b**,[10] and **6b**[4] were synthesized according to previously reported protocols.

3.1 Synthesis of 1

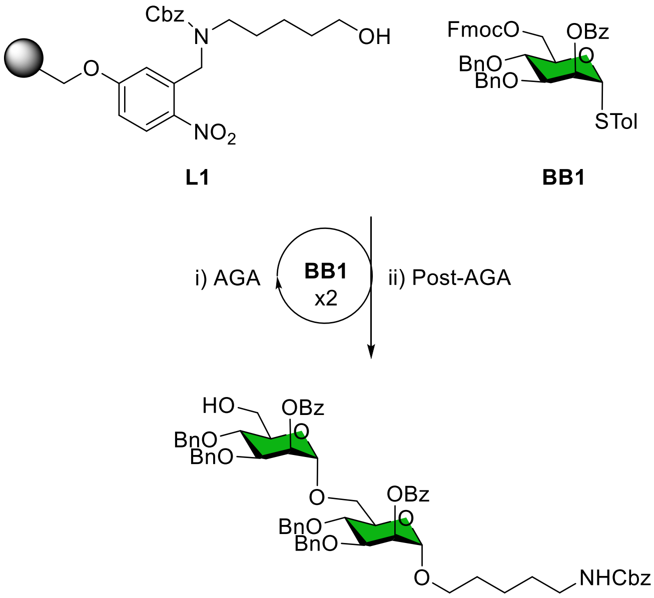

1

Step	BB	Modules	Notes
**AGA**	–	**A**	**L1** swelling
	(**BB1**)x2	(**B**, **C**, **D**, **E1**)x2	**C**: (**BB1,** −20 °C for 5 min, 0 °C for 20 min)
**Post-AGA**	–	**G1**, **I**	**I**: (method A2, t_R = 19.3 min)

Analytical data for compound **1**: ^{1}H NMR (400 MHz, $CDCl_3$) δ 8.10 (ddt, *J* = 18.6, 7.2, 1.5 Hz, 5H), 7.63–7.56 (m, 1H), 7.55–7.42 (m, 7H), 7.37–7.27 (m, 12H), 7.26–7.18 (m, 8H), 7.18–7.08 (m, 2H), 5.73 (m, 1H), 5.63 (dd, *J* = 3.3, 1.8 Hz, 1H), 5.08 (m, 3H), 4.93–4.86 (m, 3H), 4.83 (d, *J* = 11.0 Hz, 1H), 4.74 (d, *J* = 11.4 Hz, 1H), 4.62 (d, *J* = 11.0 Hz, 1H), 4.56 (d, *J* = 11.0 Hz, 1H), 4.48 (dd, *J* = 11.3, 9.8 Hz, 2H), 4.09 (dd, *J* = 9.4, 3.0 Hz, 2H), 3.99–3.88 (m, 2H), 3.86 (d, *J* = 7.7 Hz, 2H), 3.75–3.55 (m, 5H), 3.44 (m, 1H), 3.22–3.07 (m, 2H), 1.63–1.44 (m, 4H), 1.42–1.30 (m, 2H). ^{13}C NMR (101 MHz, $CDCl_3$) δ 165.9, 165.6, 156.4, 138.3, 138.2, 137.9, 137.7, 136.6, 133.3, 129.9, 129.9, 129.8, 128.6, 128.5, 128.4, 128.4, 128.4, 128.2, 128.2, 128.1, 128.1, 128.0, 128.0, 127.8, 127.8, 127.7, 127.7, 97.9, 97.8, 78.6, 77.9, 77.3, 75.2, 74.3, 73.8, 72.1, 71.6, 71.3, 70.5, 69.0, 68.7, 67.8, 66.6, 66.2, 62.0, 41.0, 29.8, 29.1, 23.4; *m/z* (HRMS+) 1152.474 $[M + Na]^+$ ($C_{67}H_{71}NNaO_{15}$ requires 1152.472). NMR data were in good agreement with those previously reported.[9]

The results obtained for the different syntheses are reported in Figure S2.

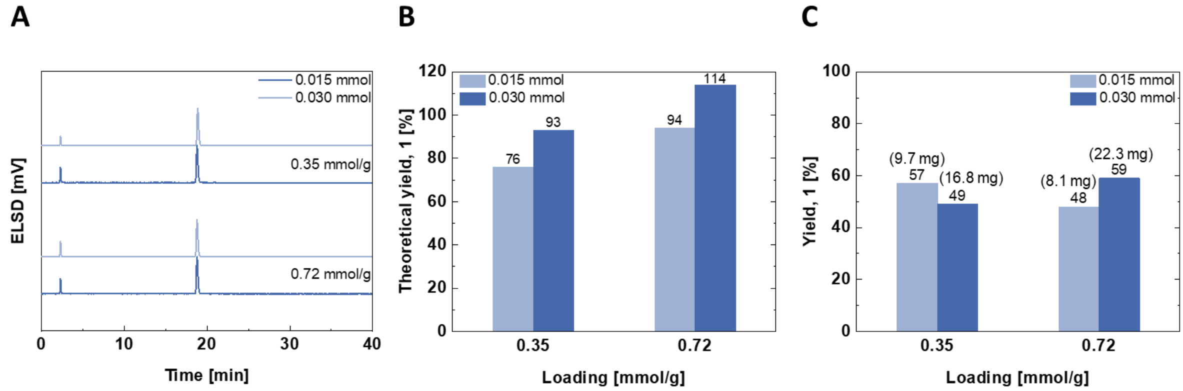

Figure S2 **A** NP-HPLC chromatograms of the crude compound **1** after AGA performed on **L1** (ELSD trace, method A1, t_R = 18.9 min). **B** Theoretical yields estimated based on the increase in resin weight after AGA. **C** Isolated yields after photocleavage and purification (method A2).

^{1}H NMR of 1 (400 MHz, $CDCl_3$)

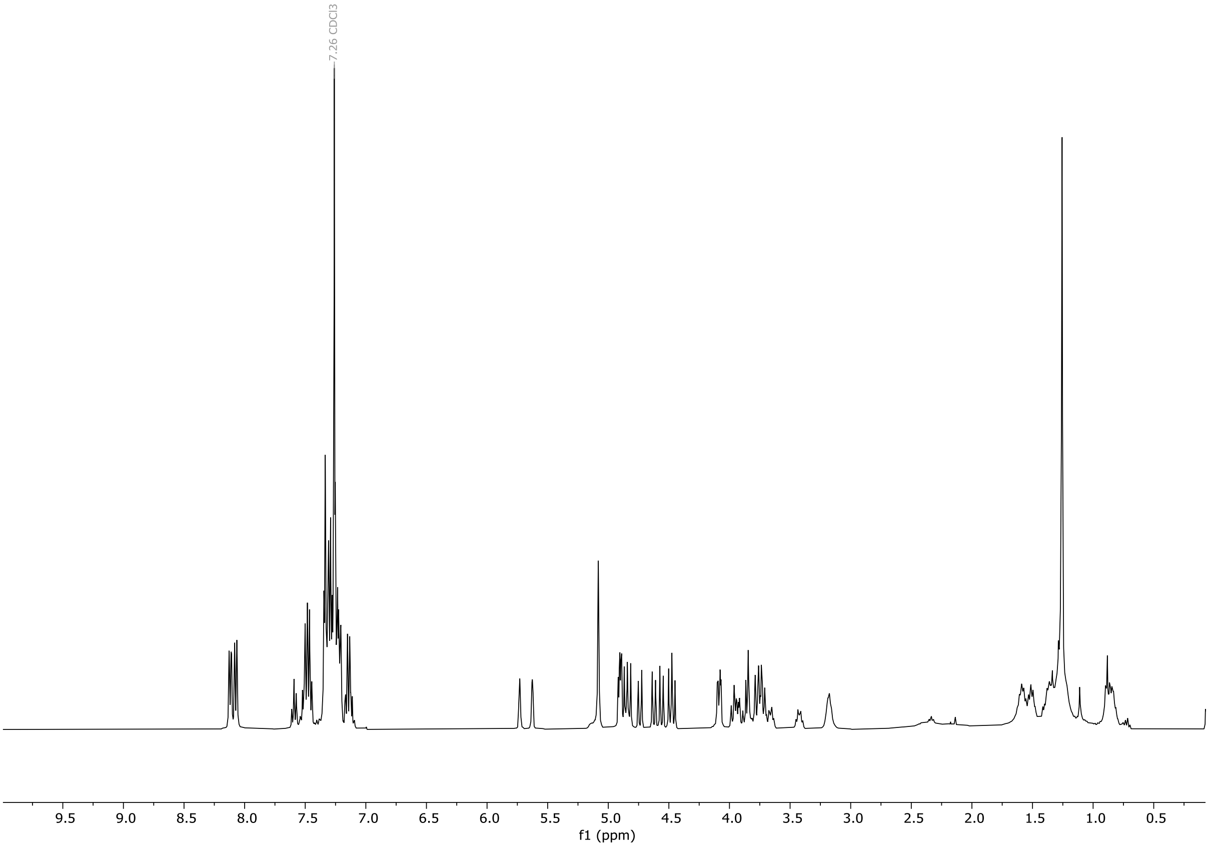

The peak at 1.25 ppm belongs to residual grease.

^{13}C NMR of 1 (101 MHz, $CDCl_3$)

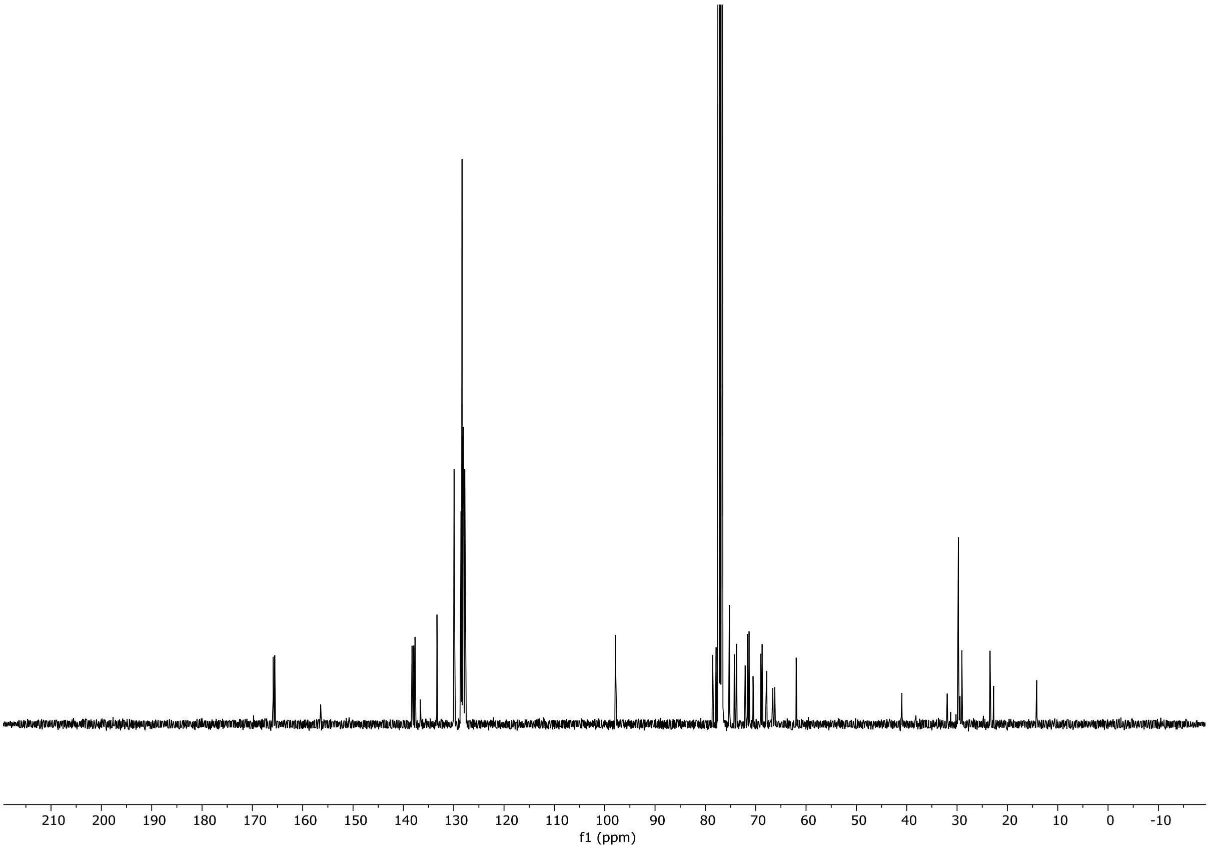

COSY NMR of 1 ($CDCl_3$)

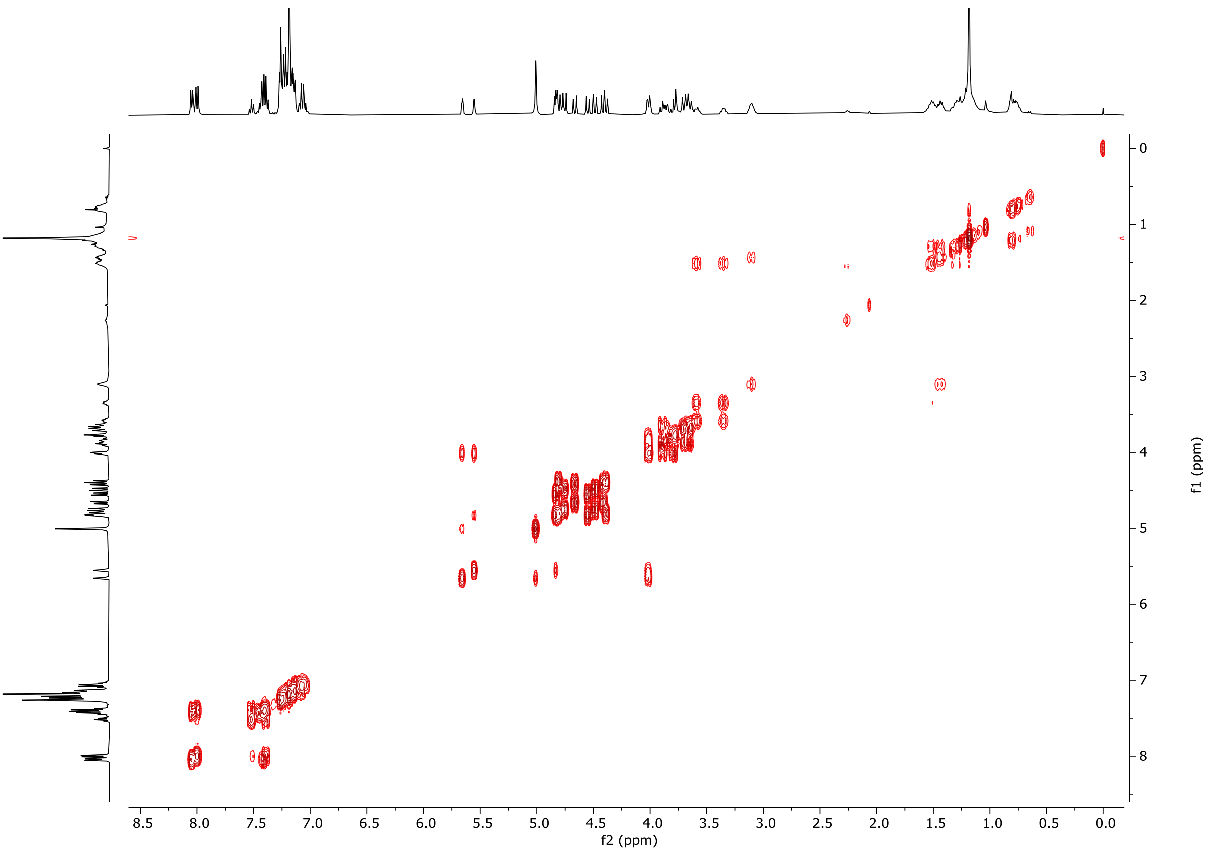

HSQC NMR of 1 ($CDCl_3$)

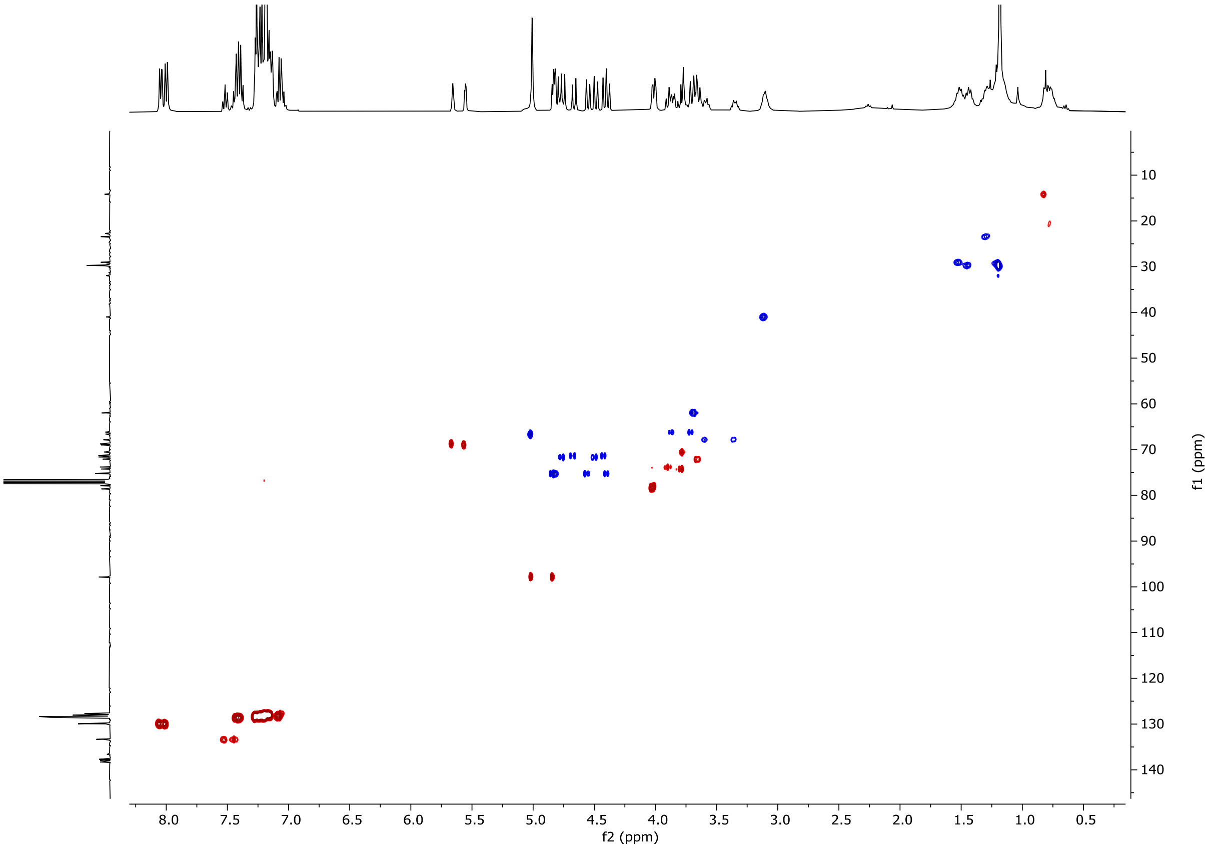

3.2 Synthesis of 2

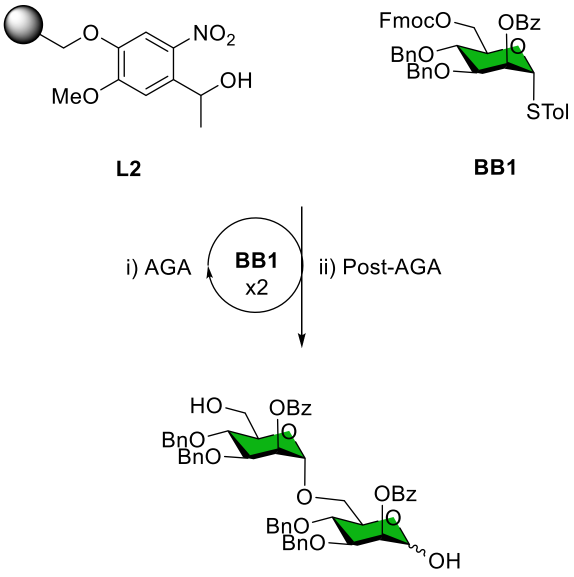

2

Step	BB	Modules	Notes
**AGA**	–	**A**	**L1** swelling
	(**BB1**)x2	(**B**, **C**, **D**, **E1**)x2	**C**: (**BB1**, −20 °C for 5 min, 0 °C for 20 min)
**Post-AGA**	–	**G1**, **I**	**I**: (method A2, t_R = 27.6 min)

Analytical data for compound **2**: ^{1}H NMR (400 MHz, $CDCl_3$) δ 8.18–8.02 (m, 5H), 7.59 (ddt, *J* = 8.8, 6.9, 1.4 Hz, 1H), 7.55–7.50 (m, 1H), 7.49 (d, *J* = 1.4 Hz, 2H), 7.48–7.46 (m, 2H), 7.45 (d, *J* = 2.0 Hz, 1H), 7.35–7.27 (m, 10H), 7.25–7.17 (m, 6H), 7.17–7.15 (m, 2H), 5.67 (ddd, *J* = 5.0, 3.2, 1.9 Hz, 2H), 5.32 (d, *J* = 1.9 Hz, 0.9H, α-H1), 5.09 (d, *J* = 1.8 Hz, 0.1H), 5.04 (d, *J* = 1.8 Hz, 0.9H), 4.95 (d, *J* = 1.2 Hz, 0.1H, β-H1), 4.89 (dd, *J* = 11.1, 8.2 Hz, 2H), 4.81 (d, *J* = 11.2 Hz, 1H), 4.74 (d, *J* = 11.4 Hz, 1H), 4.62 (d, *J* = 11.0 Hz, 1H), 4.59–4.46 (m, 3H), 4.16 (dd, *J* = 9.3, 3.3 Hz, 1H), 4.09 (m, 2H), 3.95–3.85 (m, 1H), 3.85–3.67 (m, 6H). ^{13}C NMR (101 MHz, $CDCl_3$) δ 163.3, 135.7, 135.6, 135.3, 135.2, 130.9, 130.8, 127.4, 127.3, 127.2, 126.0, 125.9, 125.8, 125.7, 125.6, 125.5, 125.4, 125.3, 125.2, 125.1, 95.6, 90.2, 75.5, 75.3, 74.8, 74.7, 74.5, 74.2, 72.7, 72.5, 71.8, 71.7, 69.4, 69.1, 68.8, 68.4, 66.7, 66.5, 65.6, 65.0, 59.8; *m/z* (HRMS+) 933.348 $[M + Na]^+$ ($C_{54}H_{54}NaO_{13}$ requires 933.346).

The results obtained for the different syntheses are reported in Figure S3.

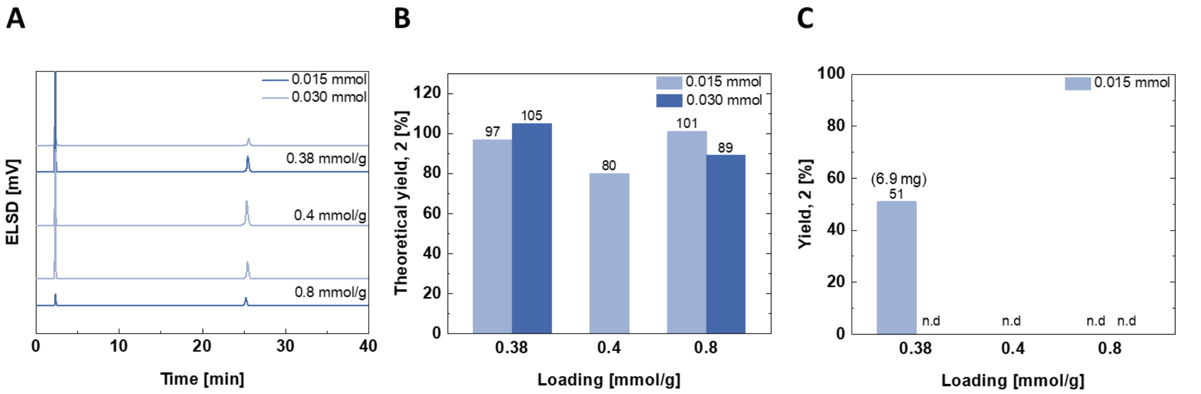

Figure S3 **A** NP-HPLC chromatograms of the crude compound **2** after AGA performed on **L2** (ELSD trace, method A1, t_R = 25.4 min). **B** Theoretical yields estimated based on the increased in resin weight after AGA. **C** Isolated yields after photocleavage and purification (method A2).

^{1}H NMR of 2 (400 MHz, $CDCl_3$)

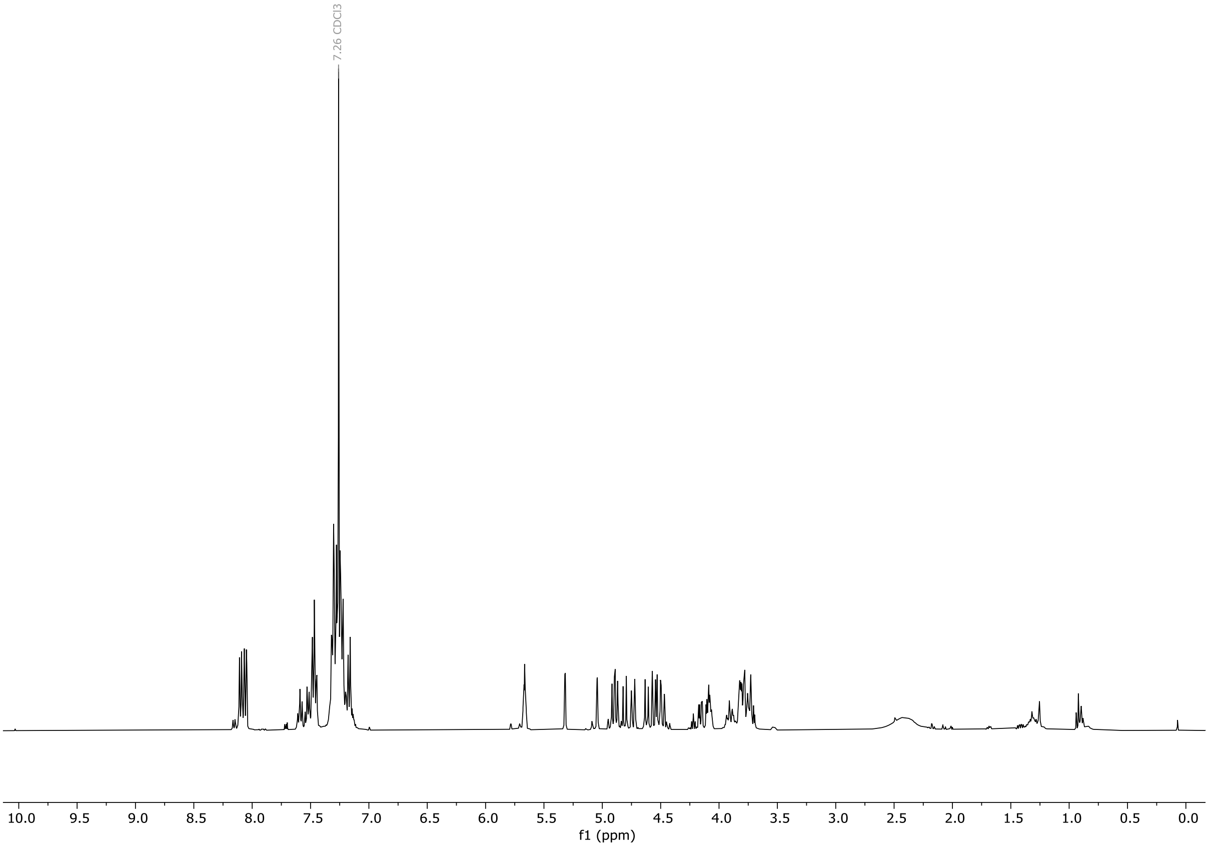

^{13}C NMR of 2 (101 MHz, $CDCl_3$)

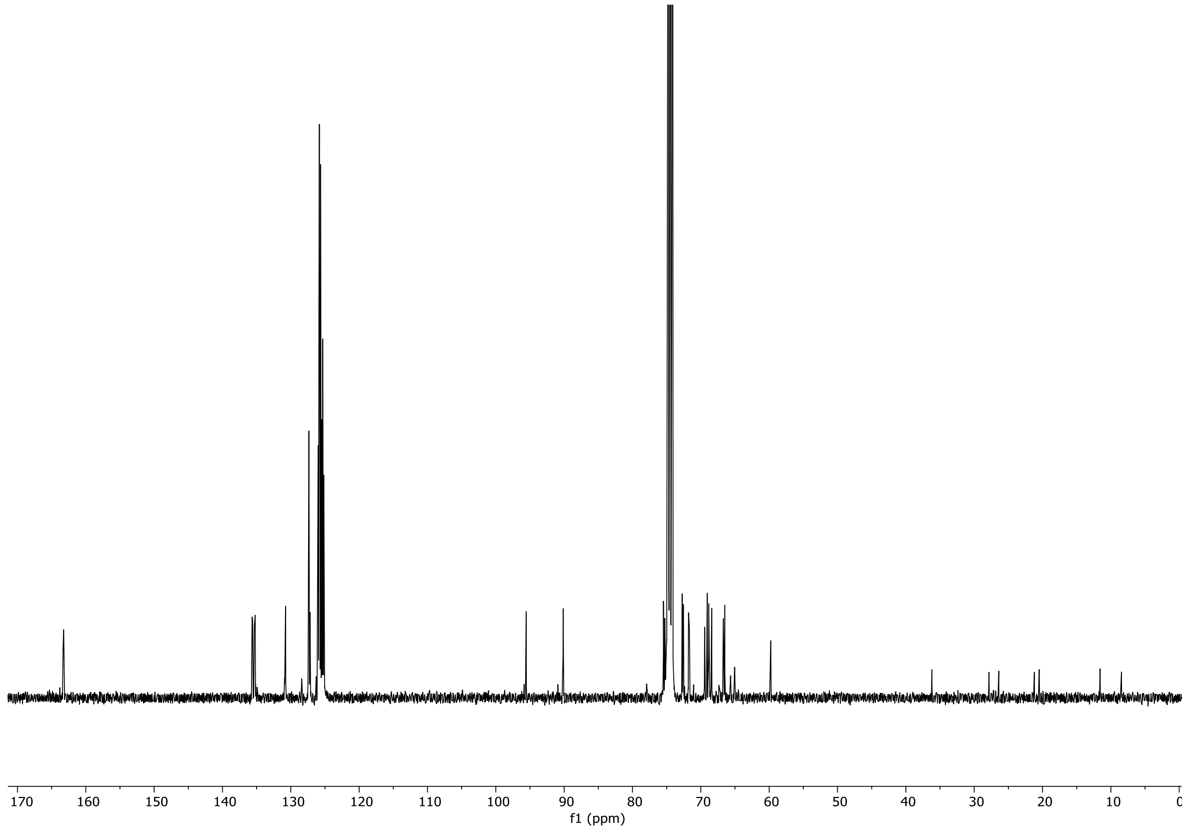

COSY NMR of 2 ($CDCl_3$)

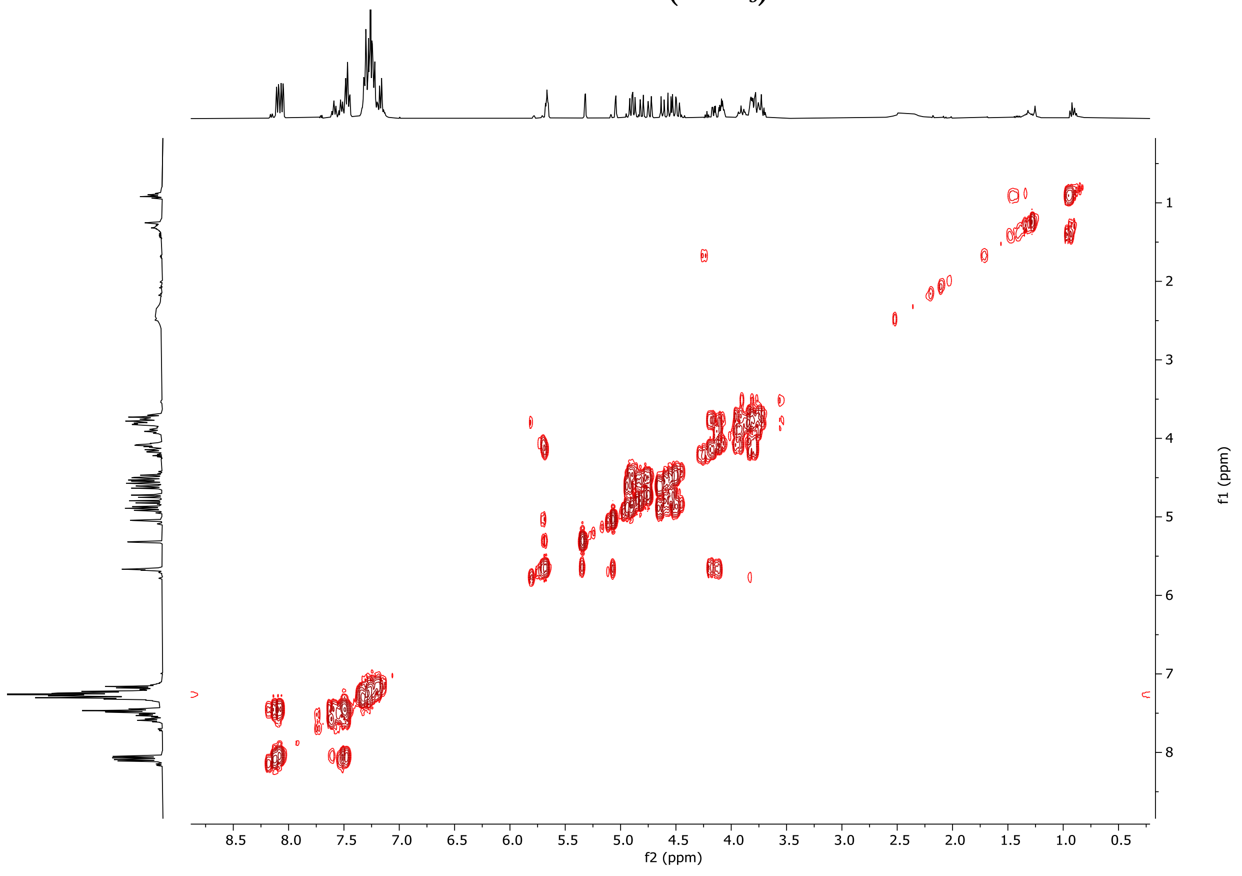

HSQC NMR of 2 ($CDCl_3$)

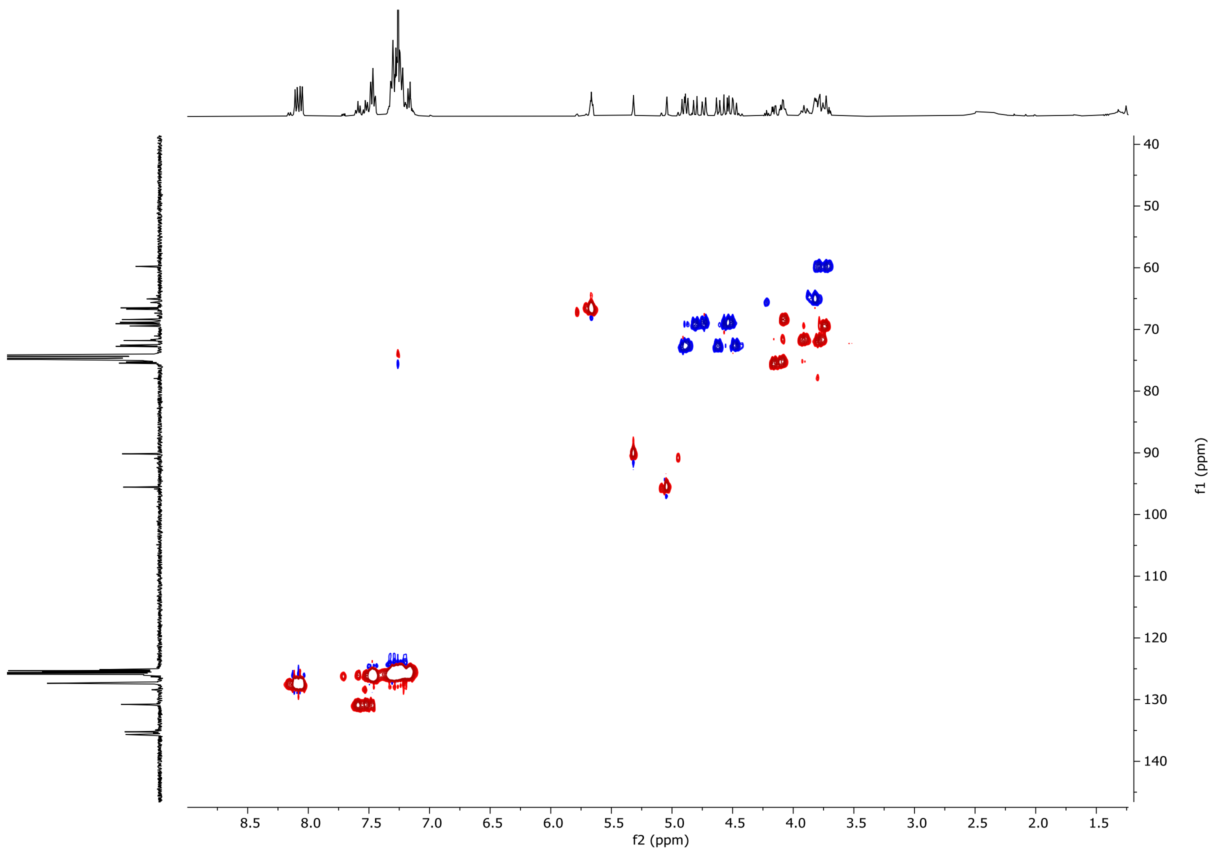

3.3 Synthesis of 3

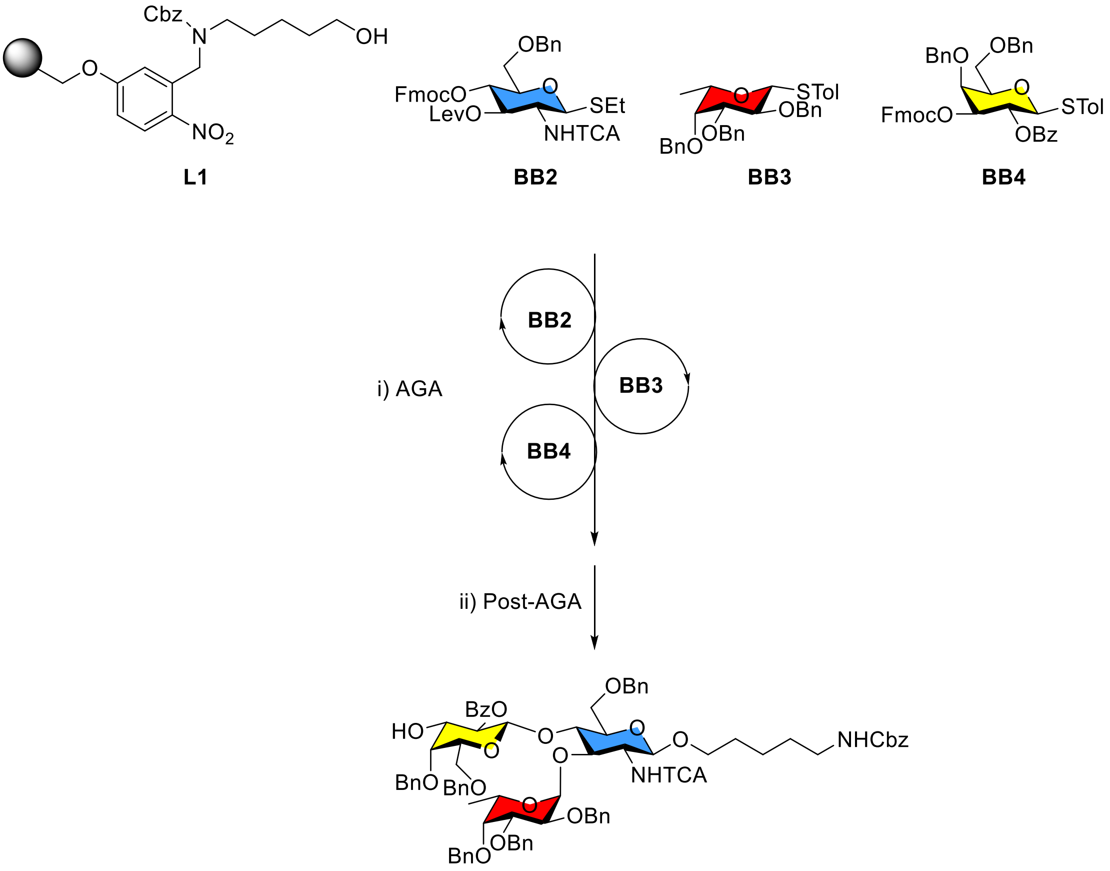

3

Step	BB	Modules	Notes
**AGA**	–	**A**	**L1** swelling
	**BB2**	(**B**, **C**, **D**, **E2**)	**C**: (**BB2**, −20 °C for 5 min, 0 °C for 40 min)
	**BB3**	(**B**, **C**, **D**, **E1**)	**C**: (**BB3**, −40 °C for 5 min, −20 °C for 20 min)
	**BB4**	(**B**, **C**, **D**, **E1**)	**C**: (**BB4**, −20 °C for 5 min, 0 °C for 20 min)
**Post-AGA**	–	**G1, I**	**I**: (method A2 , t_R = 21.8 min)

Analytical data for compound **3**: ^{1}H NMR (400 MHz, $CDCl_3$) δ 8.03–7.96 (m, 1H), 7.95–7.90 (m, 2H), 7.61–7.55 (m, 1H), 7.47–7.40 (m, 2H), 7.37–7.26 (m, 27H), 7.26–7.18 (m, 6H), 6.82 (d, J = 7.9 Hz, 1H), 5.19–5.16 (m, 1H), 5.14–5.04 (m, 3H), 4.80–4.60 (m, 7H), 4.59–4.50 (m, 2H), 4.49–4.32 (m, 4H), 4.25 (d, J = 11.4 Hz, 2H), 4.15–4.04 (m, 2H), 4.01 (dd, J = 10.2, 3.7 Hz, 1H), 3.96–3.87 (m, 2H), 3.82 (dd, J = 10.9, 3.0 Hz, 1H), 3.76–3.68 (m, 3H), 3.67–3.58 (m, 3H), 3.58 (dd, J = 10.2, 3.5 Hz, 1H), 3.51–3.45 (m, 1H), 3.42 (d, J = 1.6 Hz, 1H), 3.32–3.22 (m, 1H), 3.15–3.06 (m, 2H), 1.53–1.39 (m, 2H), 1.37–1.27 (m, 4H), 1.18 (d, J = 6.5 Hz, 3H). ^{13}C NMR (101 MHz, $CDCl_3$) δ 139.0, 129.7, 128.8, 128.6, 128.5, 128.4, 128.3, 128.2, 128.0, 127.9, 127.7, 127.6, 127.5, 127.1, 99.3, 97.3, 77.4, 77.3, 77.2, 77.1, 77.0, 76.7, 76.0, 74.9, 74.1, 73.6, 73.4, 73.2, 69.6, 66.8, 59.1, 41.2, 29.7, 29.6, 23.1, 16.6; *m/z* (HRMS+) 1517.507 [M + Na]$^+$ ($C_{82}H_{89}Cl_3N_2NaO_{18}$ requires 1517.507).

The results obtained for the different syntheses are reported in Figure S4.

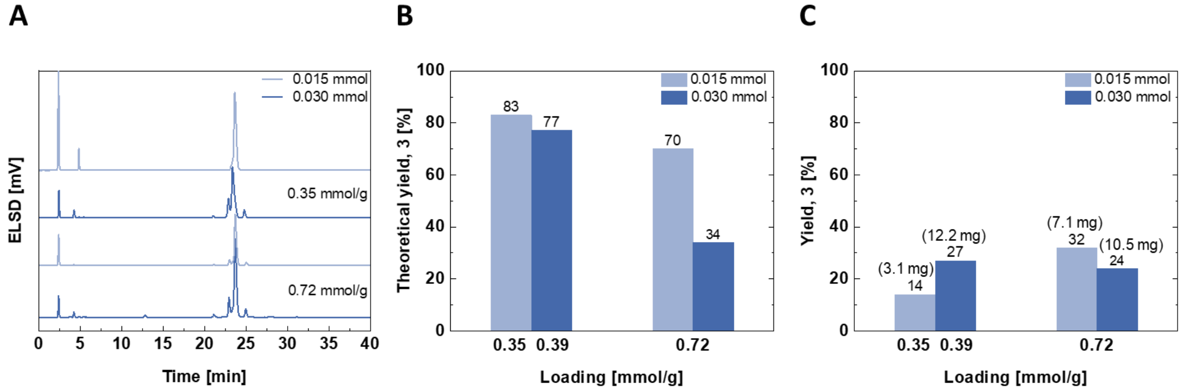

Figure S4 **A** NP-HPLC chromatograms of the crude compound **3** after AGA performed on **L1** (ELSD trace, method A1, t_R = 23.8 min). **B** Theoretical yields estimated based on the increased in resin weight after AGA. **C** Isolated yields after photocleavage and purification (method A2).

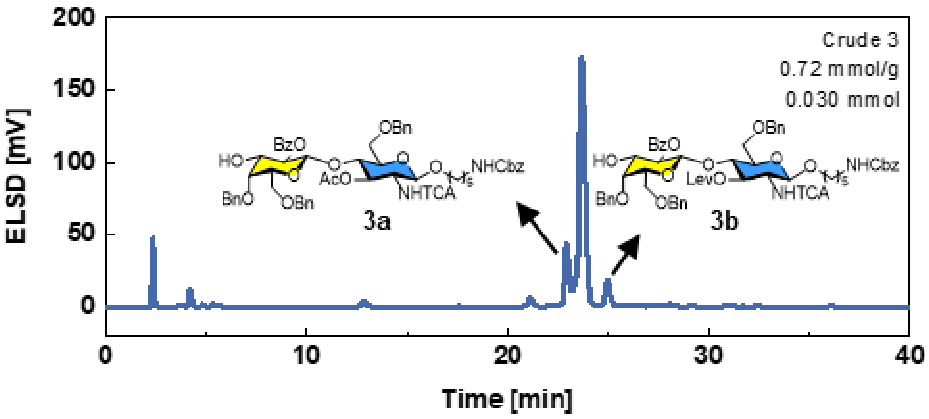

Figure S5 Representative NP-HPLC chromatogram of the crude compound **3** after AGA for the synthesis performed on 0.03 mmol scale on **L1** with a loading of 0.72 mmol/g (ELSD trace, method A1). Deletion sequences **3a** (t_R = 22.8 min) and **3b** (t_R = 25.0 min) were assigned after MALDI-TOF MS analysis. *m/z* (MALDI-TOF MS) of compound **3a** 1143.0 [M + Na]$^+$ ($C_{57}H_{63}Cl_3N_2NaO_{15}$ requires 1143.3). *m/z* (MALDI-TOF MS) of compound **3b** 1199.0 [M + Na]$^+$ ($C_{82}H_{89}Cl_3N_2NaO_{18}$ requires 1199.3).

^{1}H NMR of 3 (400 MHz, $CDCl_3$)

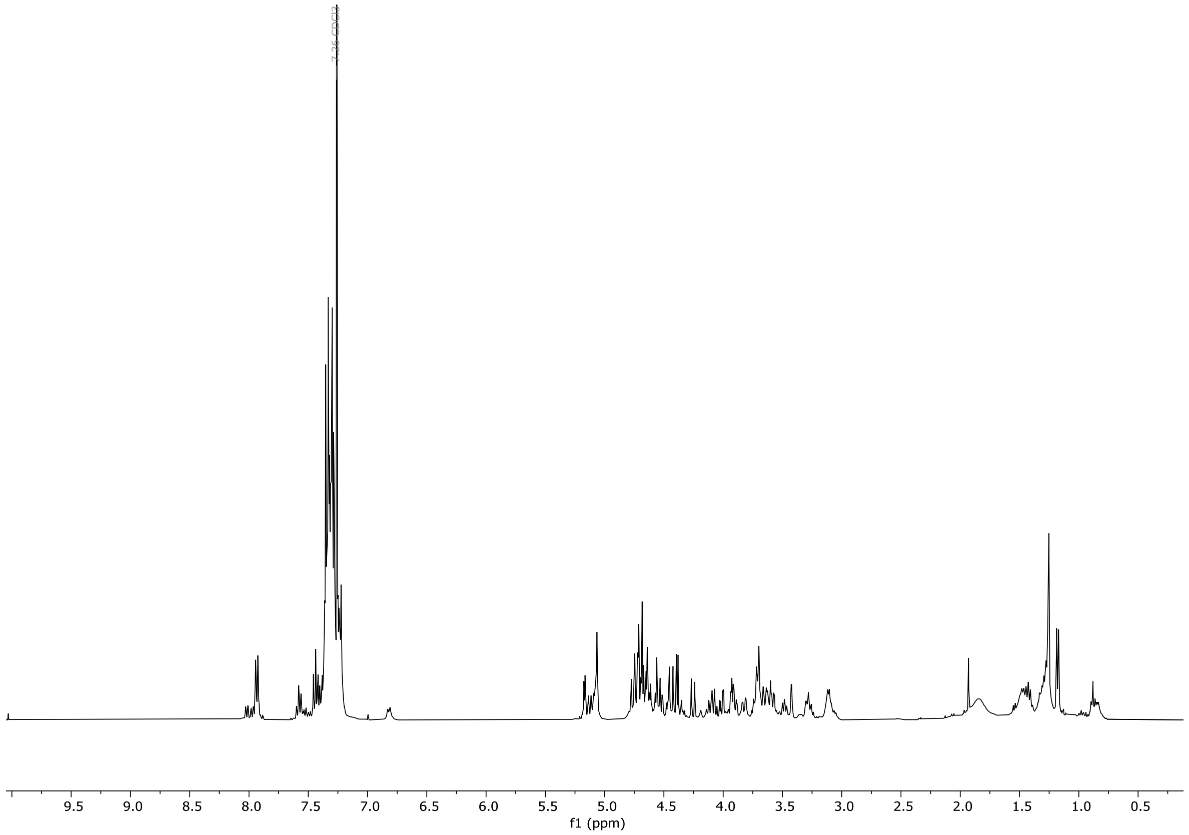

The peak at 0.87 ppm belongs to residual grease.

^{13}C NMR of 3 (101 MHz, $CDCl_3$)

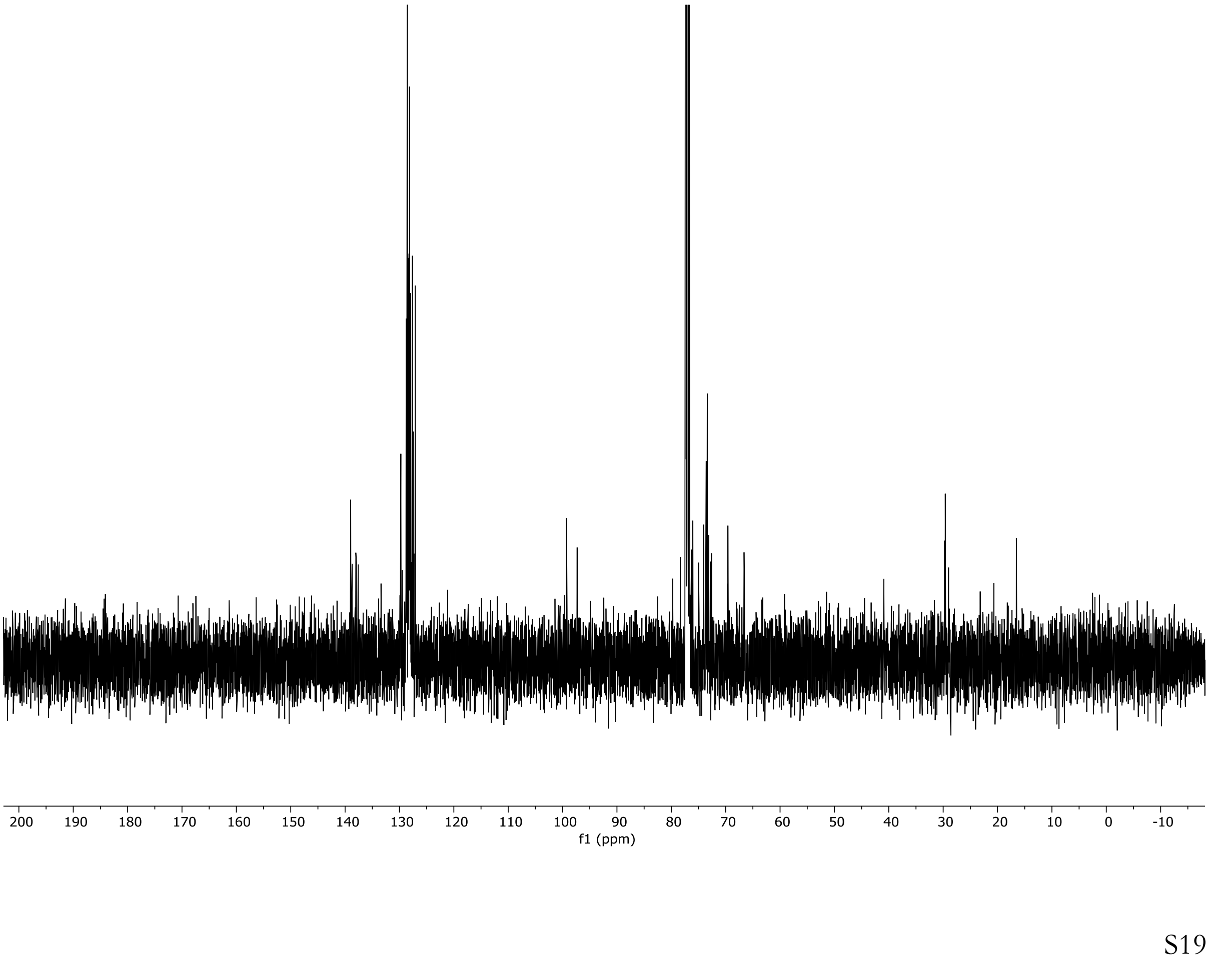

COSY NMR of 3 ($CDCl_3$)

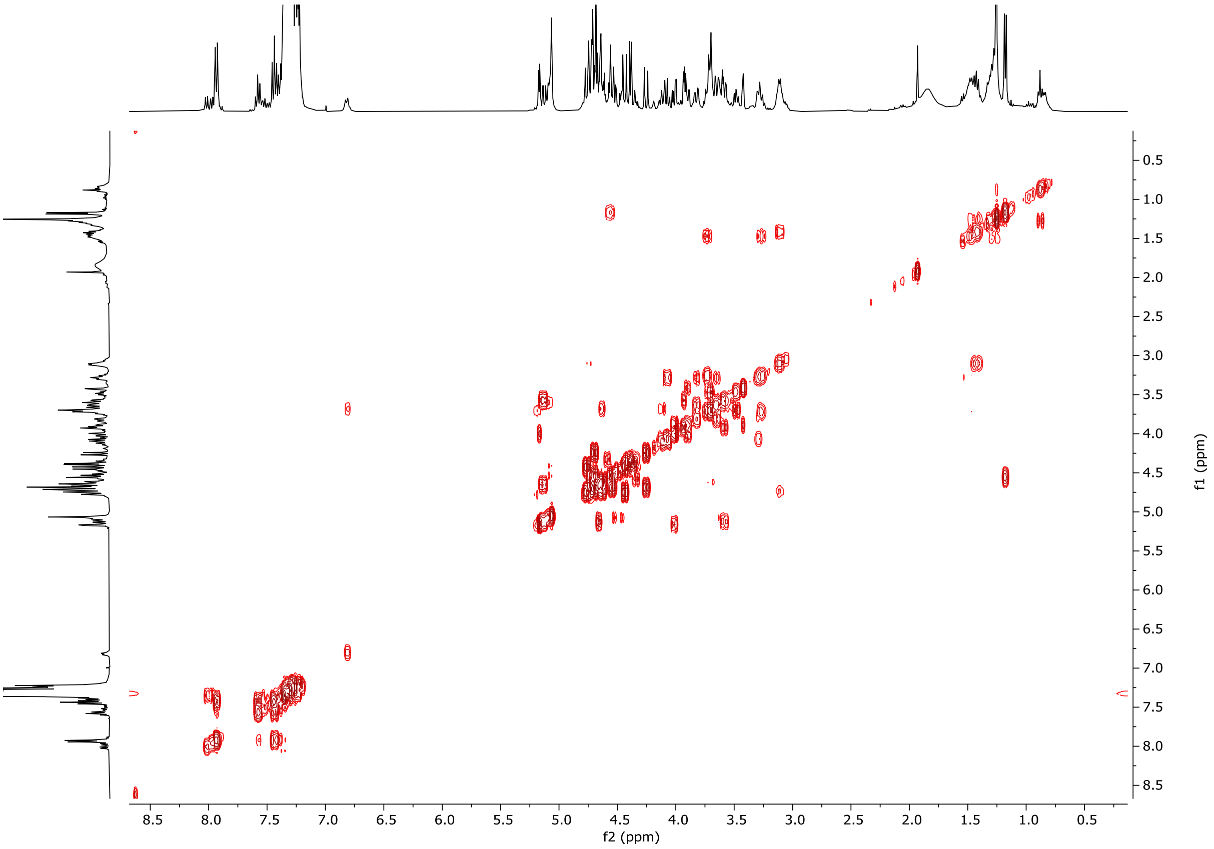

HSQC NMR of 3 ($CDCl_3$)

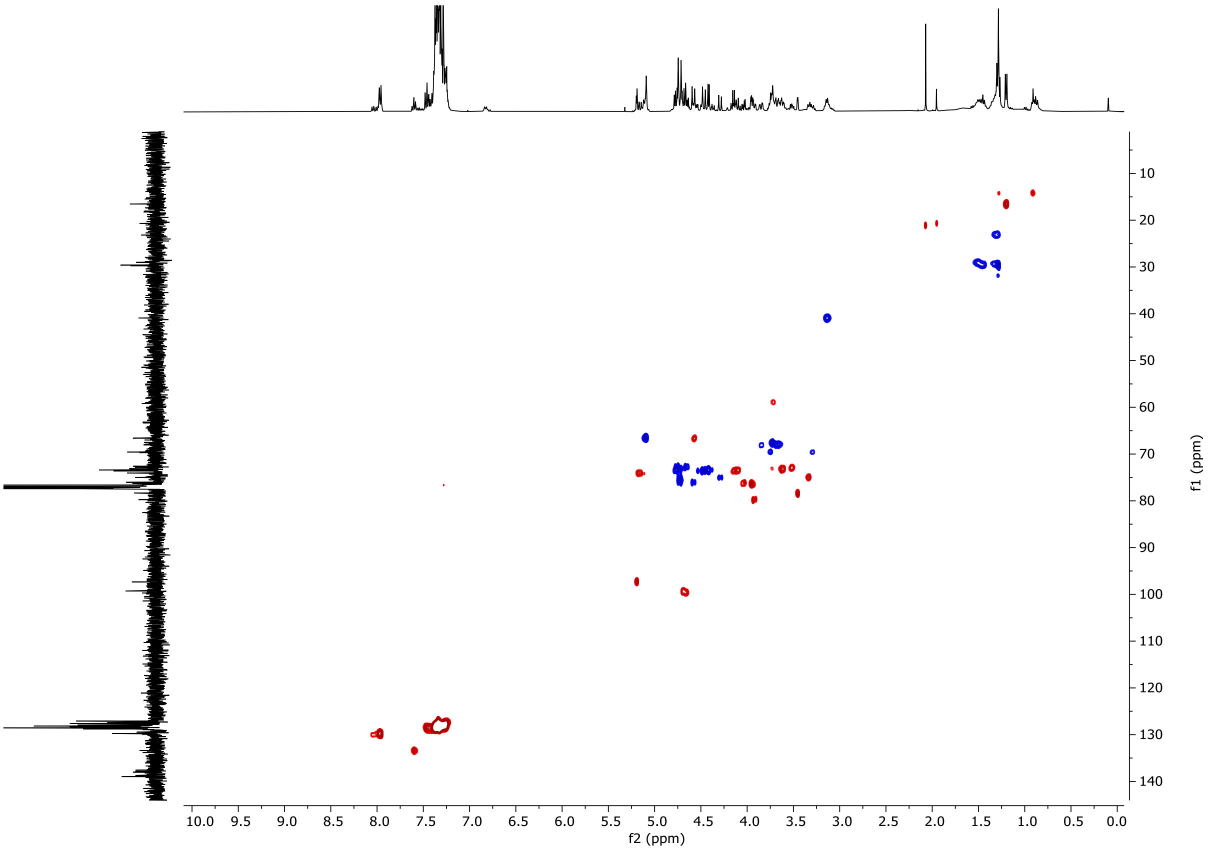

3.4 Synthesis of 4

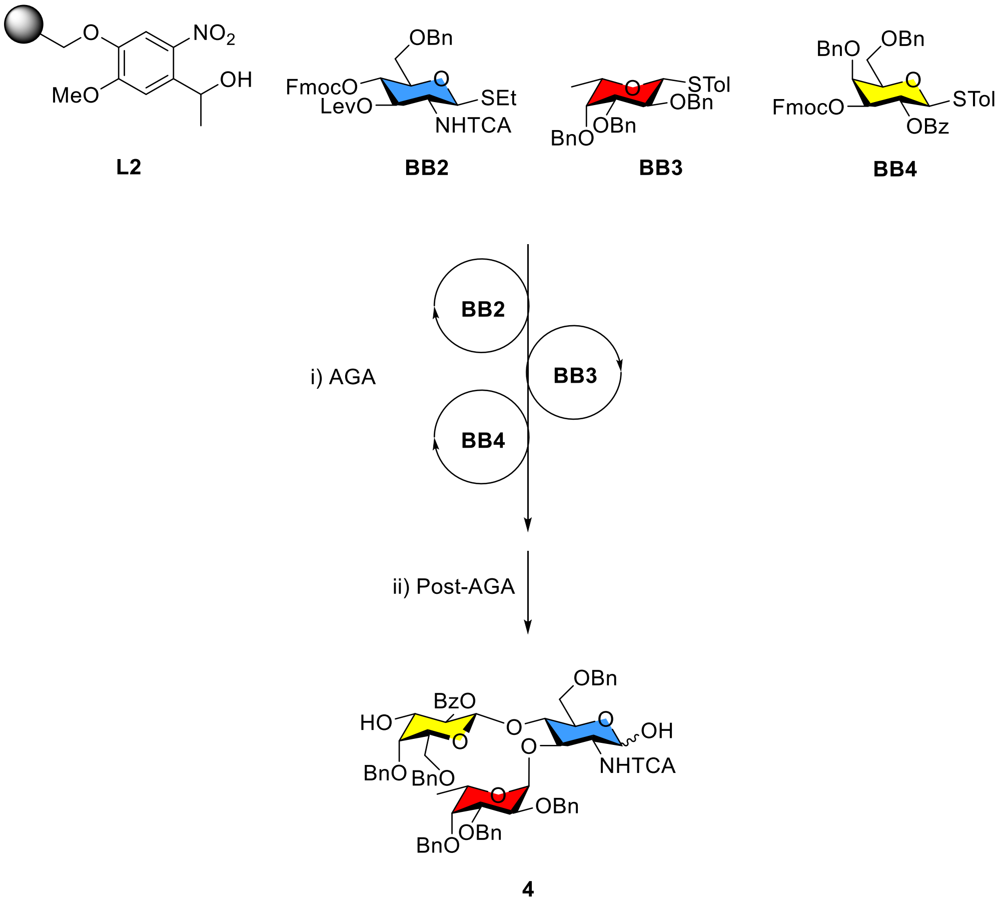

Step	BB	Modules	Notes
AGA	–	**A**	**L1** swelling
	**BB2**	(**B**, **C**, **D**, **E2**)	**C**: (**BB2**, −20 °C for 5 min, 0 °C for 40 min)
	**BB3**	(**B**, **C**, **D**, **E1**)	**C**: (**BB3**, −40 °C for 5 min, −20 °C for 20 min)
	**BB4**	(**B**, **C**, **D**, **E1**)	**C**: (**BB4**, −20 °C for 5 min, 0 °C for 20 min)
**Post-AGA**	–	**G1**, **I**	**I**: (method A2, t_R = 27.2 min)

Analytical data for compound **4**: ^{1}H NMR (600 MHz, $CDCl_3$) δ 7.97–7.93 (m, 2H), 7.62–7.59 (m, 1H), 7.49–7.45 (m, 2H), 7.42–7.28 (m, 21H), 7.27–7.20 (m, 8H), 6.94 (d, *J* = 9.4 Hz, 1H), 5.31 (d, *J* = 3.7 Hz, 1H), 5.24 (dd, *J* = 3.9 Hz, 3.9 Hz, 1H, α-H1), 5.16 (dd, *J* = 10.1, 8.0 Hz, 1H), 4.80 (dd, *J* = 11.8, 7.8 Hz, 2H), 4.72 (dd, *J* = 11.3, 6.3 Hz, 3H), 4.66–4.54 (m, 5H), 4.44 (d, *J* = 12.2 Hz, 1H), 4.40 (d, *J* = 11.8 Hz, 1H), 4.36–4.31 (m, 2H), 4.27 (d, *J* = 11.4 Hz, 1H), 4.18–4.11 (m, 2H), 4.04 (dd, *J* = 10.2, 3.7 Hz, 1H), 3.95–3.92 (m, 2H), 3.89 (dd, *J* = 11.0, 3.1 Hz, 1H), 3.84–3.80 (m, 1H), 3.75 (d, *J* = 7.0 Hz, 2H), 3.59–3.53 (m, 2H), 3.51–3.46 (m, 2H), 1.23 (d, *J* = 6.5 Hz, 3H). ^{13}C NMR (101 MHz, $CDCl_3$) δ 166.2, 161.7, 139.2, 139.0, 138.4, 138.2, 138.1, 137.7, 137.6, 137.5, 133.4, 129.8, 129.7, 129.5, 129.0, 128.8, 128.7, 128.6, 128.5, 128.4, 128.3, 128.2, 128.1, 128.0, 127.9, 127.8, 127.7, 127.6, 127.5, 127.4, 127.3, 127.0, 99.5, 97.5, 97.1, 92.5, 90.8,

79.3, 78.5, 77.3, 76.4, 76.2, 75.9, 75.8, 75.0, 74.1, 73.7, 73.5, 73.4, 73.3, 73.2, 73.0, 72.9, 72.7, 72.5, 71.8, 70.6, 67.8, 67.5, 66.7, 55.9, 16.6; *m*/*z* (HRMS+) 1298.385 [M + Na]$^+$ ($C_{69}H_{72}Cl_3NNaO_{16}$ requires 1298.381).

The results obtained for the different syntheses are reported in Figure S6.

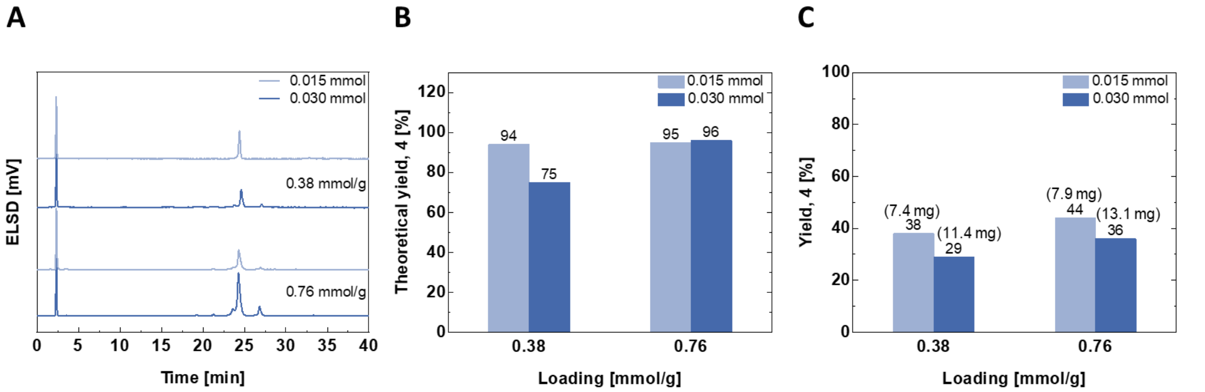

Figure S6 A NP-HPLC chromatograms of the crude compound **4** after AGA performed on **L2** (ELSD trace, method A1, t_R = 25.9 min). **B** Theoretical yields estimated based on the increased in resin weight after AGA. **C** Isolated yields after photocleavage and purification (method A2).

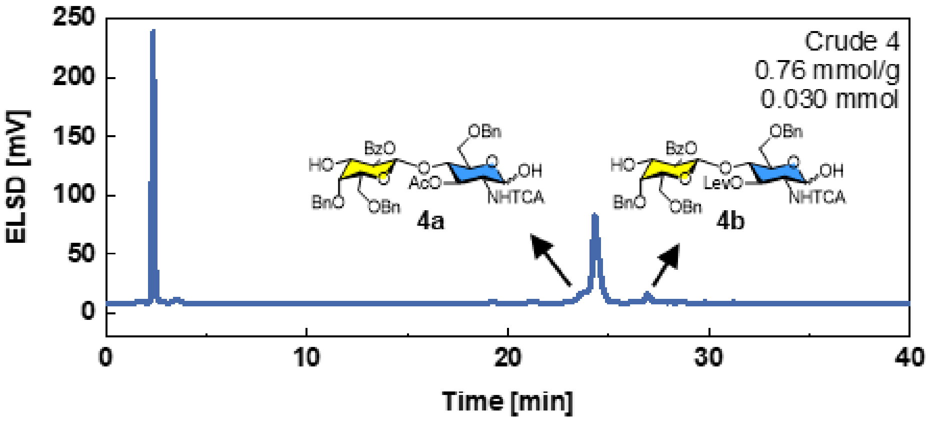

Figure S7 Representative NP-HPLC chromatogram of the crude compound **4** after AGA for the synthesis performed on 0.03 mmol scale on **L2** with a loading of 0.76 mmol/g (ELSD trace, method A1). Deletion sequences **4a** (t_R = 23.9 min) and **4b** (t_R = 27.0 min) were assigned by MALDI-TOF MS. *m*/*z* (MALDI-TOF MS) of compound **4a** 924.0 [M + Na]$^+$ ($C_{44}H_{46}Cl_3NNaO_{13}$ requires 924.2). *m*/*z* (MALDI-TOF MS) of compound **4b** 979.9 [M + Na]$^+$ ($C_{47}H_{50}Cl_3NNaO_{14}$ requires 980.2).

^{1}H NMR of 4 (600 MHz, $CDCl_3$)

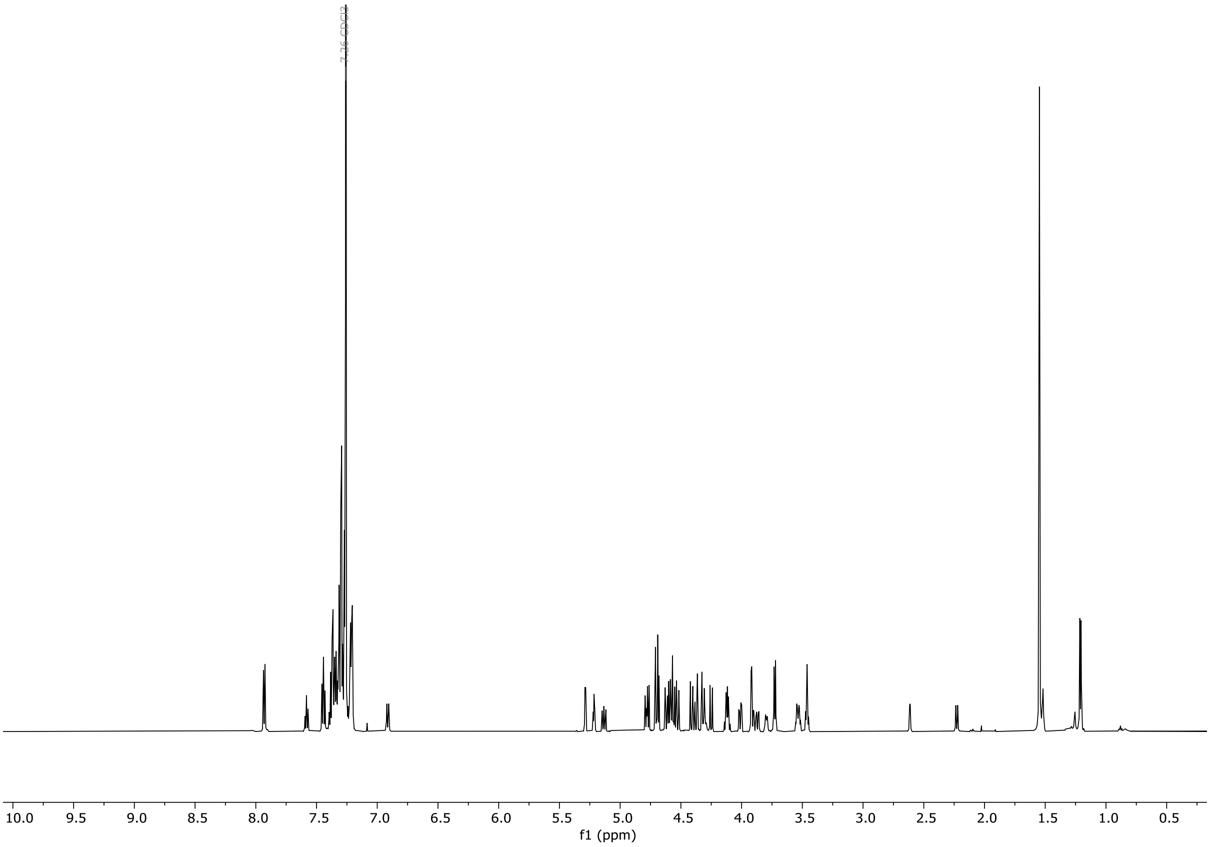

^{13}C NMR of 4 (101 MHz, $CDCl_3$)

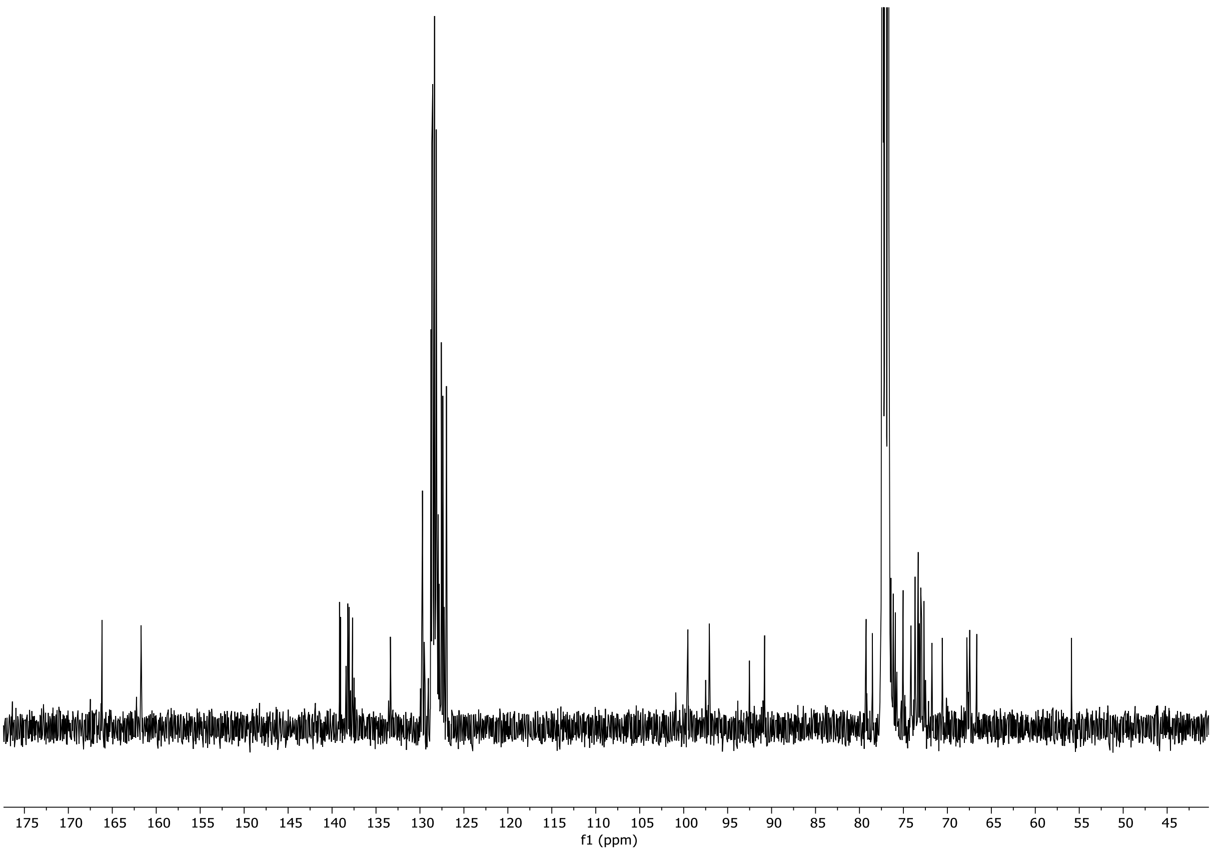

COSY NMR of 4 ($CDCl_3$)

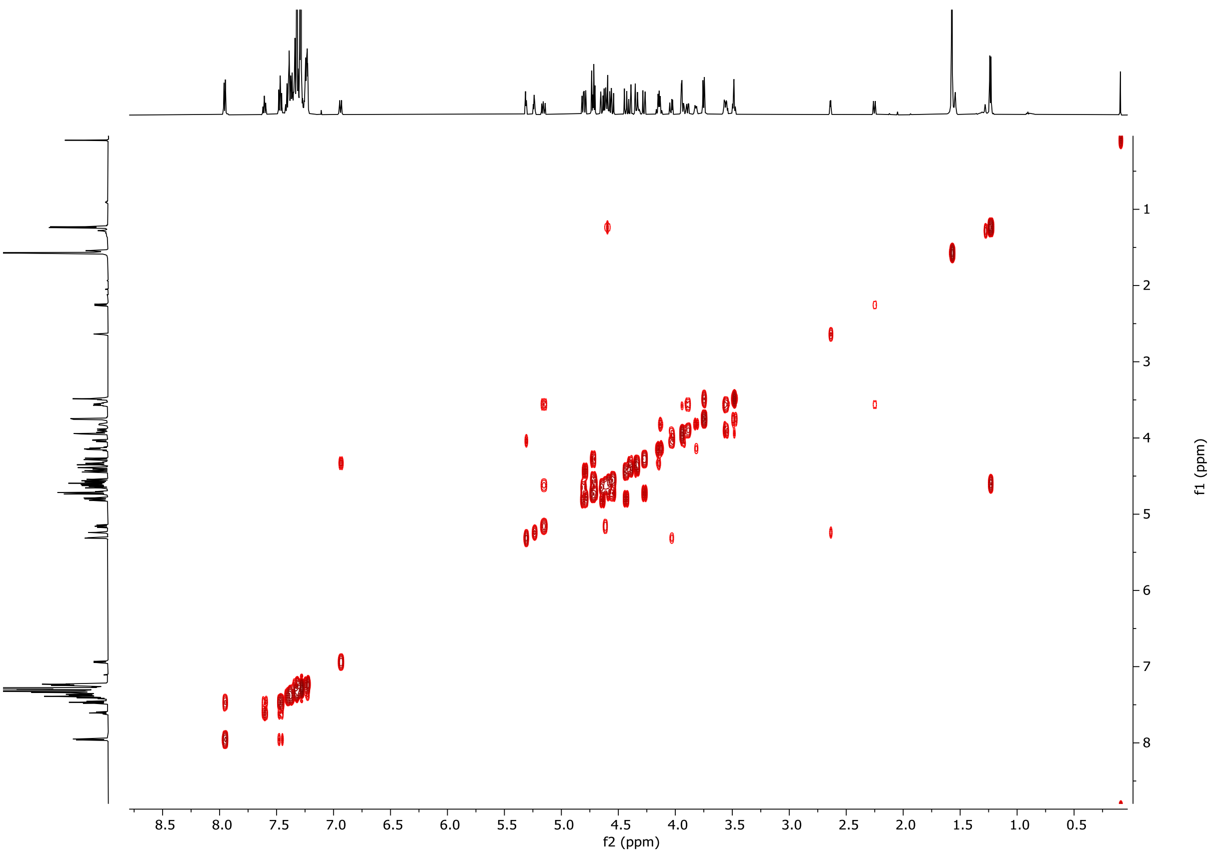

HSQC NMR of 4 ($CDCl_3$)

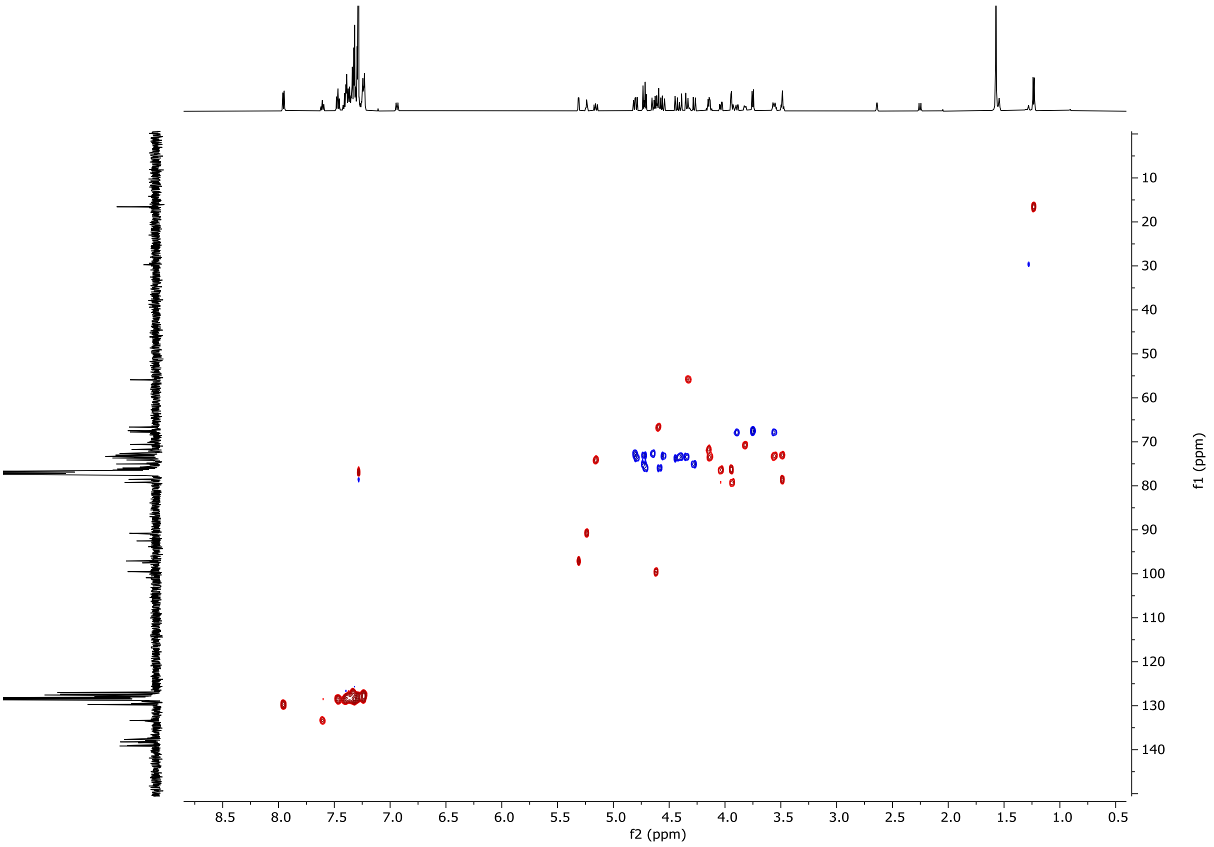

3.5 Synthesis of 5

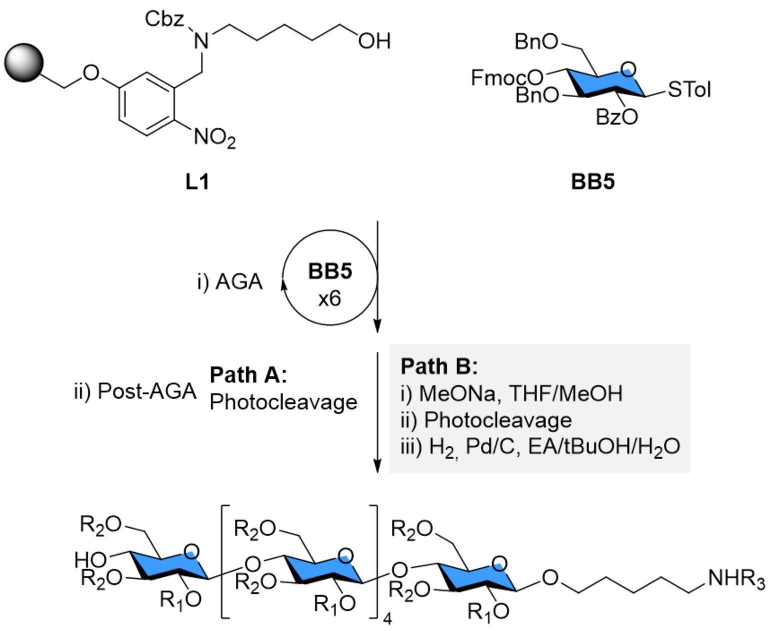

5a R_1 = Bz, R_2 = Bn, R_3 = Cbz
5b R_1 = R_2 = R_3 = H

Step	BB	Modules	Notes
**AGA**	–	**A**	**L1** swelling
	(**BB5**)x6	(**B**, **C**, **D**, **E1**)x6	**C**: (**BB5**, −20 °C for 5 min, 0 °C for 20 min)
**Post-AGA (5a)**	–	**G1**, **I**	**I**: (method A2, t_R = 27.7 min)
**Post-AGA (5b)**		**F**, **G1**, **H**, **I**	**I**: (method C)

Analytical data for compound **5a**: ^{1}H NMR (400 MHz, $CDCl_3$) δ 8.10 (s, 1H), 7.93–7.86 (m, 4H), 7.86–7.80 (m, 7H), 7.65–7.51 (m, 5H), 7.50–7.38 (m, 10H), 7.38–7.30 (m, 11H), 7.30–7.22 (m, 10H), 7.18–7.03 (m, 35H), 7.00–6.84 (m, 12H), 5.26–5.05 (m, 6H), 5.05–5.02 (m, 2H), 4.96–4.81 (m, 6H), 4.77–4.66 (m, 2H), 4.64 (d, *J* = 8.1 Hz, 1H), 4.60–4.47 (m, 9H), 4.45–4.42 (m, 1H), 4.42–4.37 (m, 4H), 4.36 (d, *J* = 6.1 Hz, 1H), 4.31–4.23 (m, 5H), 4.15 (dd, *J* = 11.9 Hz, 11.9 Hz, 2H), 4.08 (dd, *J* = 9.3 Hz, 9.3 Hz, 1H), 4.04–3.86 (m, 6H), 3.82–3.77 (dd, *J* = 9.0 Hz, 9.0 Hz, 1H), 3.74–3.67 (m, 2H), 3.63–3.25 (m, 14H), 3.24–3.18 (m, 2H), 3.16–3.06 (m, 1H), 2.91–2.73 (m, 6H), 1.45–1.30 (m, 2H), 1.24–1.14 (m, 2H), 1.15–0.99 (m, 2H). ^{13}C NMR (101 MHz, $CDCl_3$) δ 165.2, 165.1, 165.0, 164.9, 156.4, 139.0, 134.0, 138.9, 138.8, 138.3, 138.6, 137.9, 137.8, 137.8, 137.7, 136.9, 133.5, 133.3, 133.2, 133.1, 133.0, 130.2, 129.9, 129.8, 129.7, 128.6, 128.5, 128.4, 128.3, 128.2, 128.1, 128.0, 127.9, 127.8, 127.7, 127.2, 127.1, 127.0, 101.3, 100.2, 100.1, 100.0, 99.9, 82.0, 80.2, 80.1, 80.0, 77.3, 77.2, 77.0, 76.3, 76.2, 76.0, 74.8, 74.7, 74.6, 74.5, 74.4, 74.3, 74.2, 74.1, 73.9, 73.7, 73.6, 73.5, 73.4, 73.2, 73.1, 71.3, 69.5, 67.6, 67.5, 67.2, 66.6, 40.6, 29.5, 28.9, 23.2; *m/z* (HRMS+) 2937.160 $[M + Na]^+$ ($C_{175}H_{175}NO_{39}Na$ requieres 2937.163).

Analytical data for compound **5b**: ^{1}H NMR (400 MHz, D_2O) δ 4.57–4.53 (m, 4H), 4.53–4.49 (m, 2H), 4.00 (dd, *J* = 12.4, 2.0 Hz, 4H), 3.98–3.92 (m, 3H), 3.84 (td, *J* = 11.7, 11.3, 5.1 Hz, 5H), 3.75 (dt, *J* = 11.8, 5.4 Hz, 2H), 3.73–3.62 (m, 13H), 3.55–3.49 (m, 3H), 3.46–3.41 (m, 2H), 3.41–3.36 (m, 4H), 3.33 (dt, *J* = 9.1,

7.3 Hz, 2H), 3.04–3.01 (m, 2H), 1.71 (tt, J = 13.7, 7.1 Hz, 4H), 1.48 (t, J = 7.7 Hz, 2H). ^{13}C NMR (101 MHz, D_2O) δ 102.5, 102.4, 102.3, 102.3, 102.1, 101.9, 79.0, 78.5, 78.3, 78.2, 77.0, 75.9, 75.4, 74.8, 74.7, 74.3, 74.0, 73.9, 74.0, 73.2, 73.1, 73.1, 72.9, 72.9, 70.0, 69.5, 69.4, 60.5, 59.9, 59.8, 59.8, 58.9, 39.3, 28.1, 26.4, 22.0; m/z (HRMS+) 1076.424 [M + H]$^+$ ($C_{41}H_{74}NO_{31}$ requires 1076.424); NMR data were in good agreement with those previously reported.[10]

The results obtained for the different syntheses are reported in Figure S8.

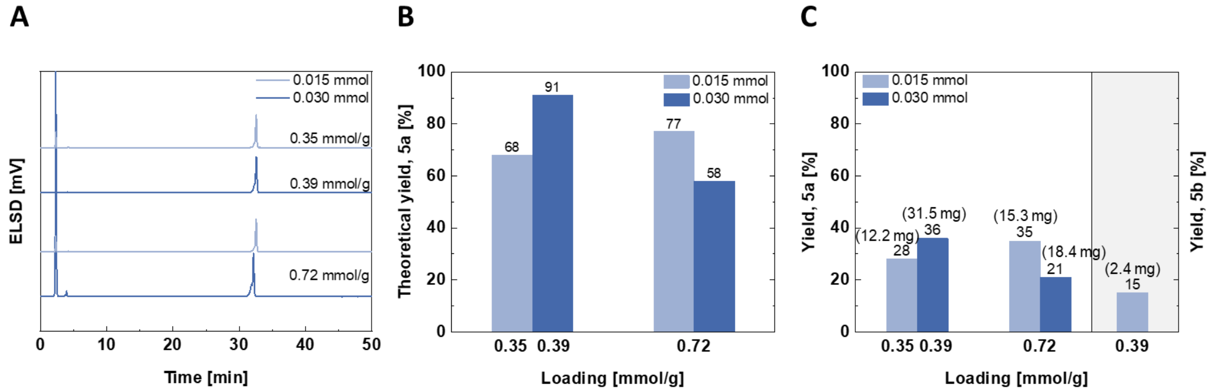

Figure S8 **A** NP-HPLC chromatograms of the crude compound **5a** after AGA performed on **L1** (ELSD trace, method A1, t_R = 32.7 min). **B** Theoretical yields estimated based on the increased in resin weight after AGA. **C** Isolated yields after post AGA for **5a** (path A) and **5b** (path B).

^{1}H NMR of 5a (400 MHz, $CDCl_3$)

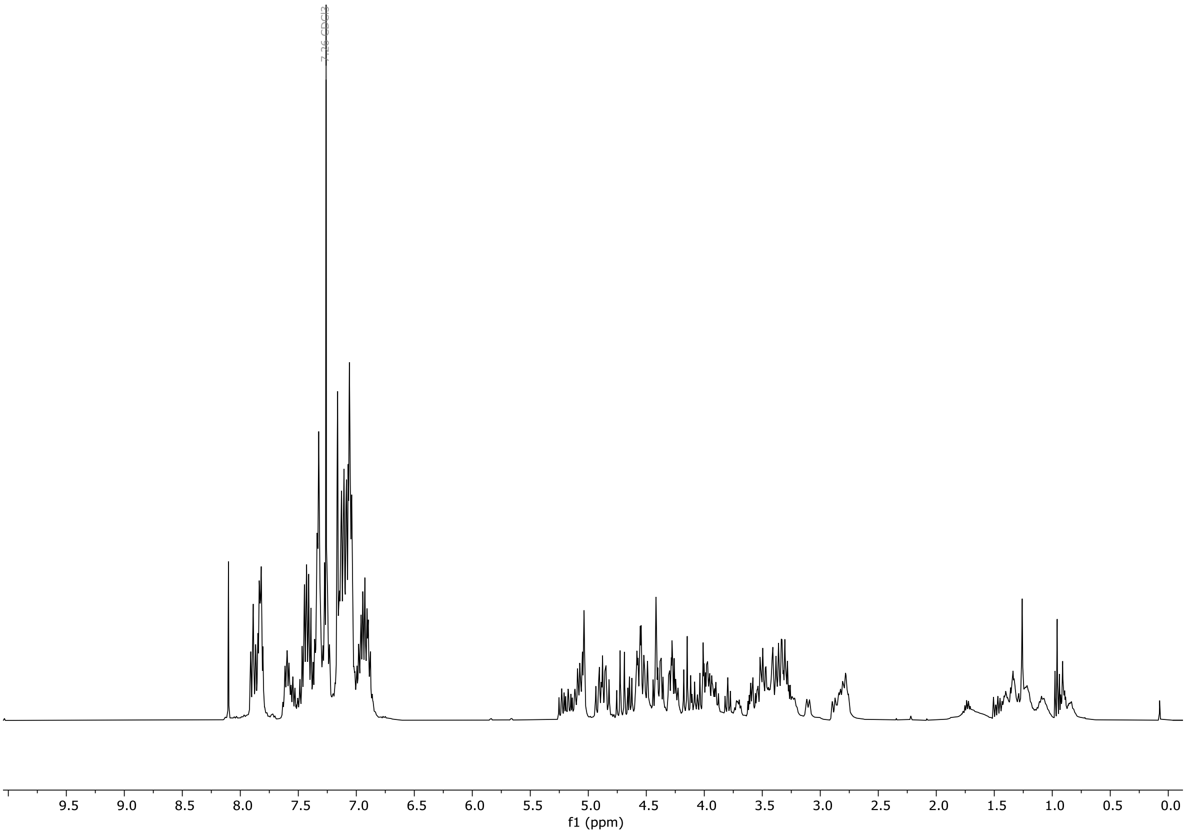

The peak at 1.25 ppm belongs to residual grease.

^{13}C NMR of 5a (101 MHz, $CDCl_3$)

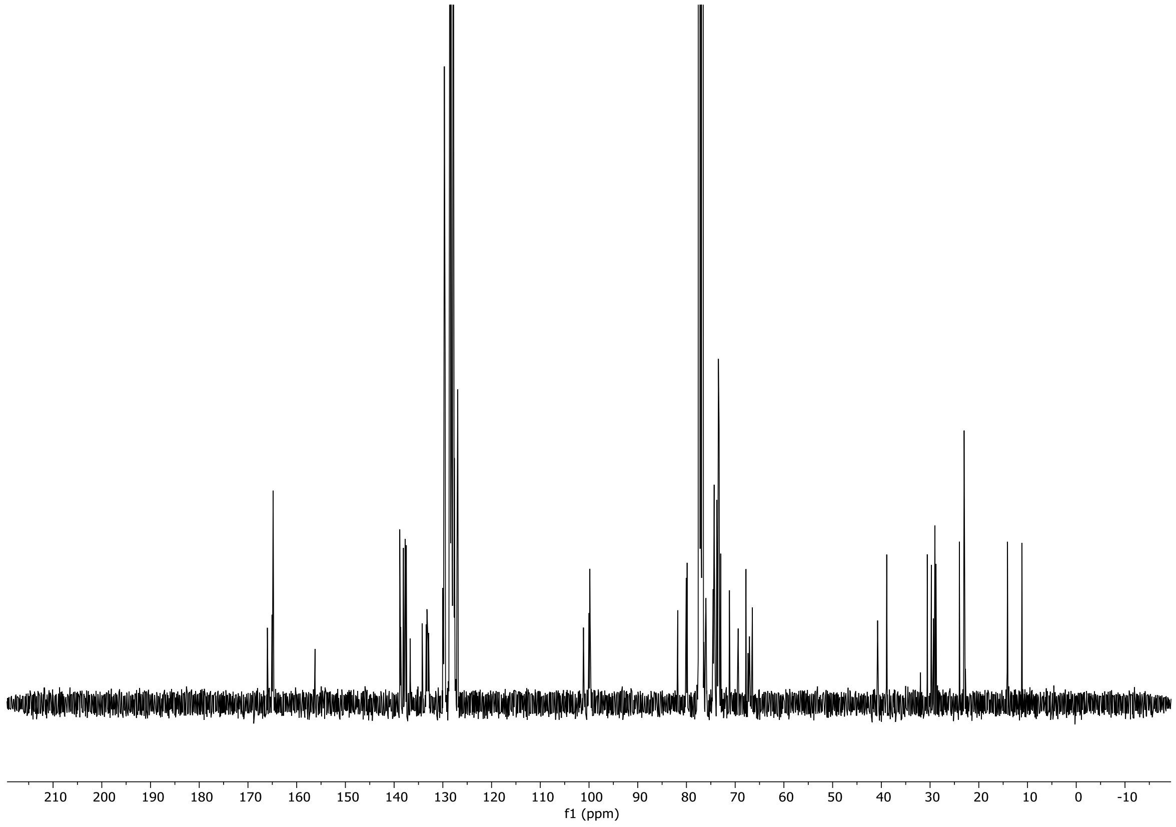

COSY NMR of 5a ($CDCl_3$)

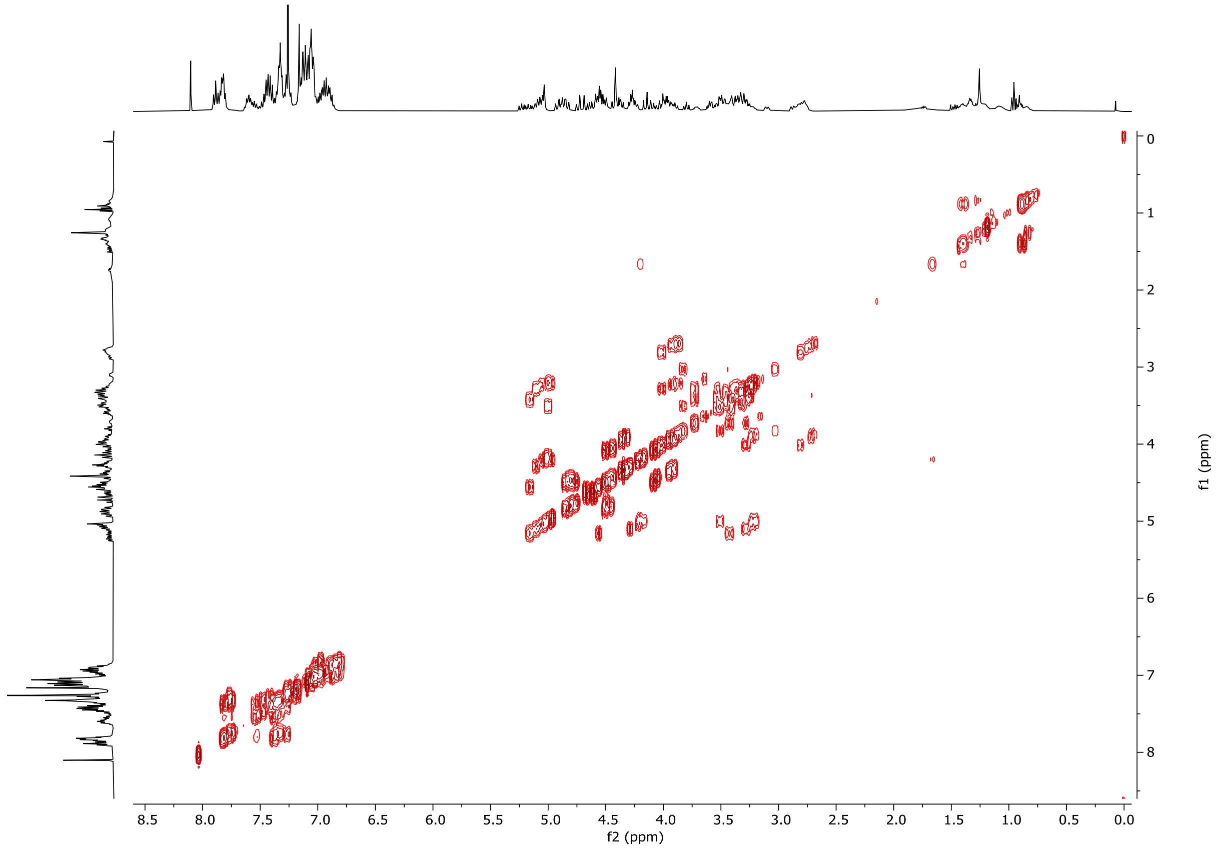

HSQC NMR of 5a ($CDCl_3$)

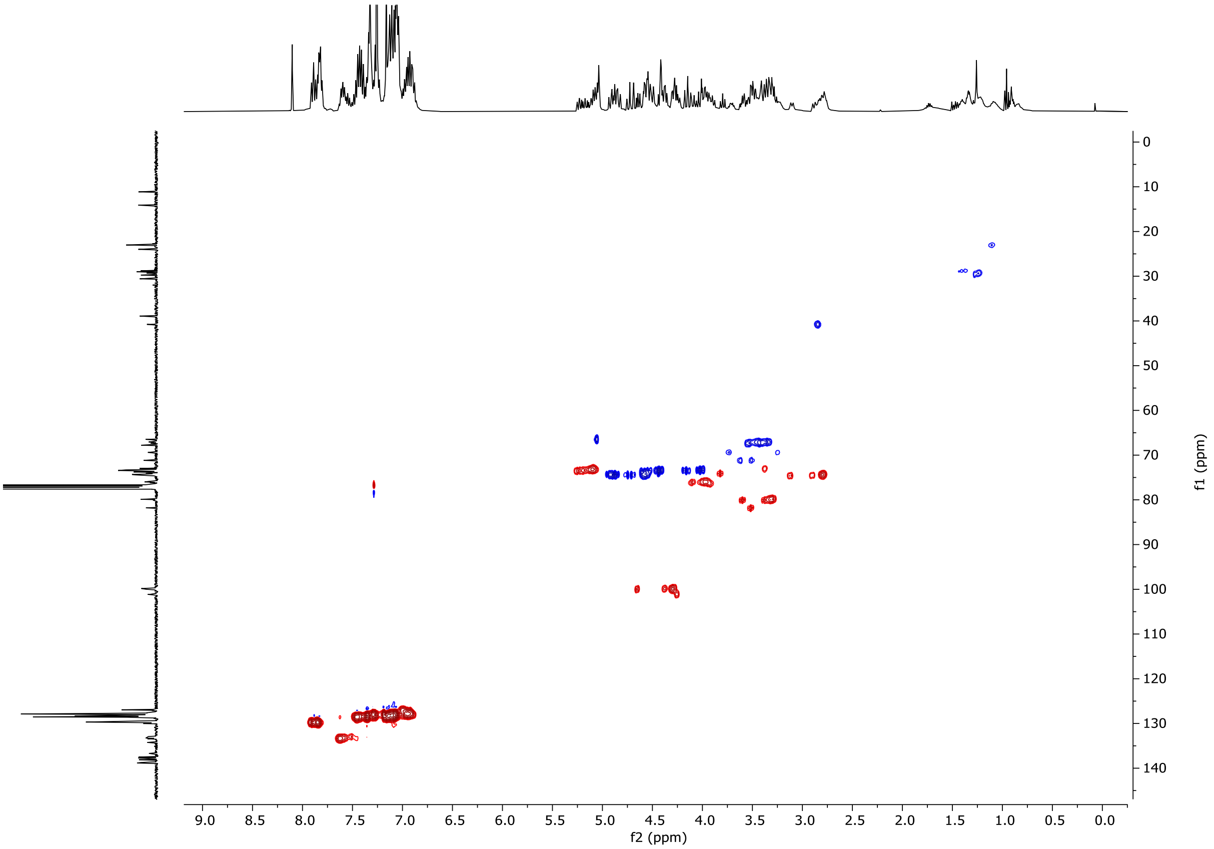

^{1}H NMR of 5b (400 MHz, $CDCl_3$)

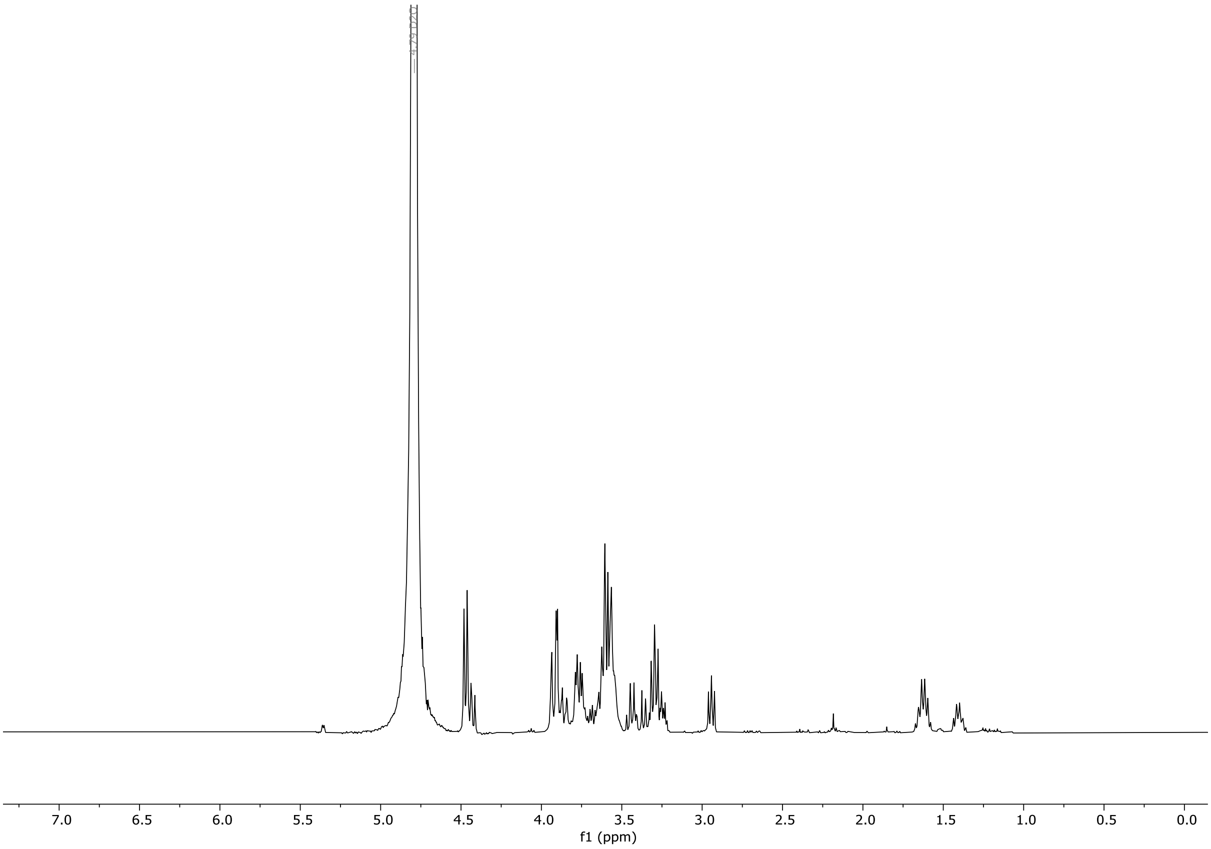

^{13}C NMR of 5b (101 MHz, $CDCl_3$)

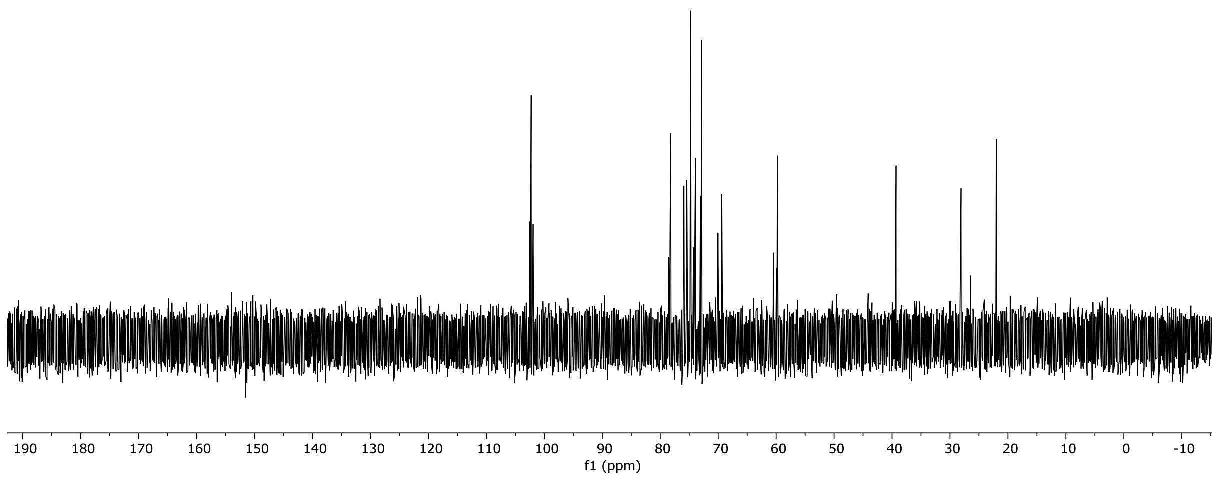

COSY NMR of 5b ($CDCl_3$)

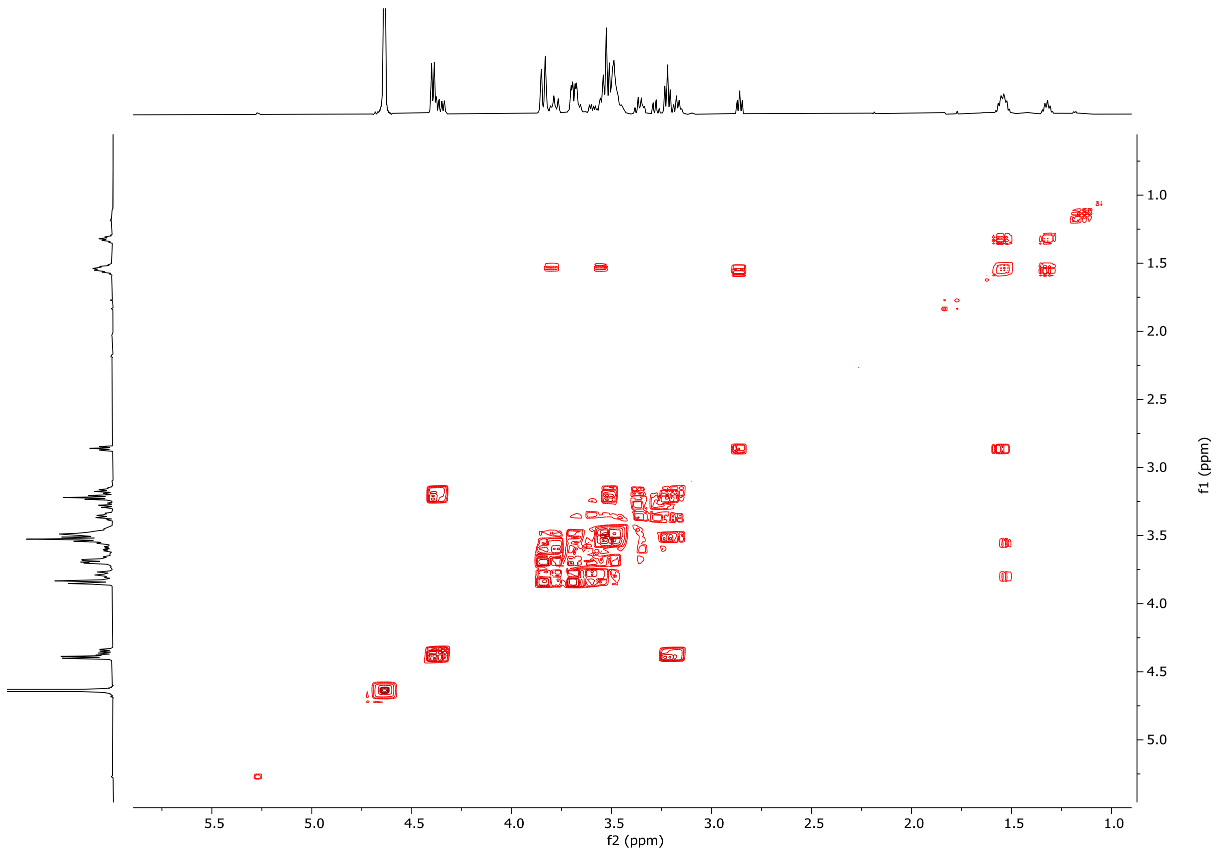

HSQC NMR of 5b ($CDCl_3$)

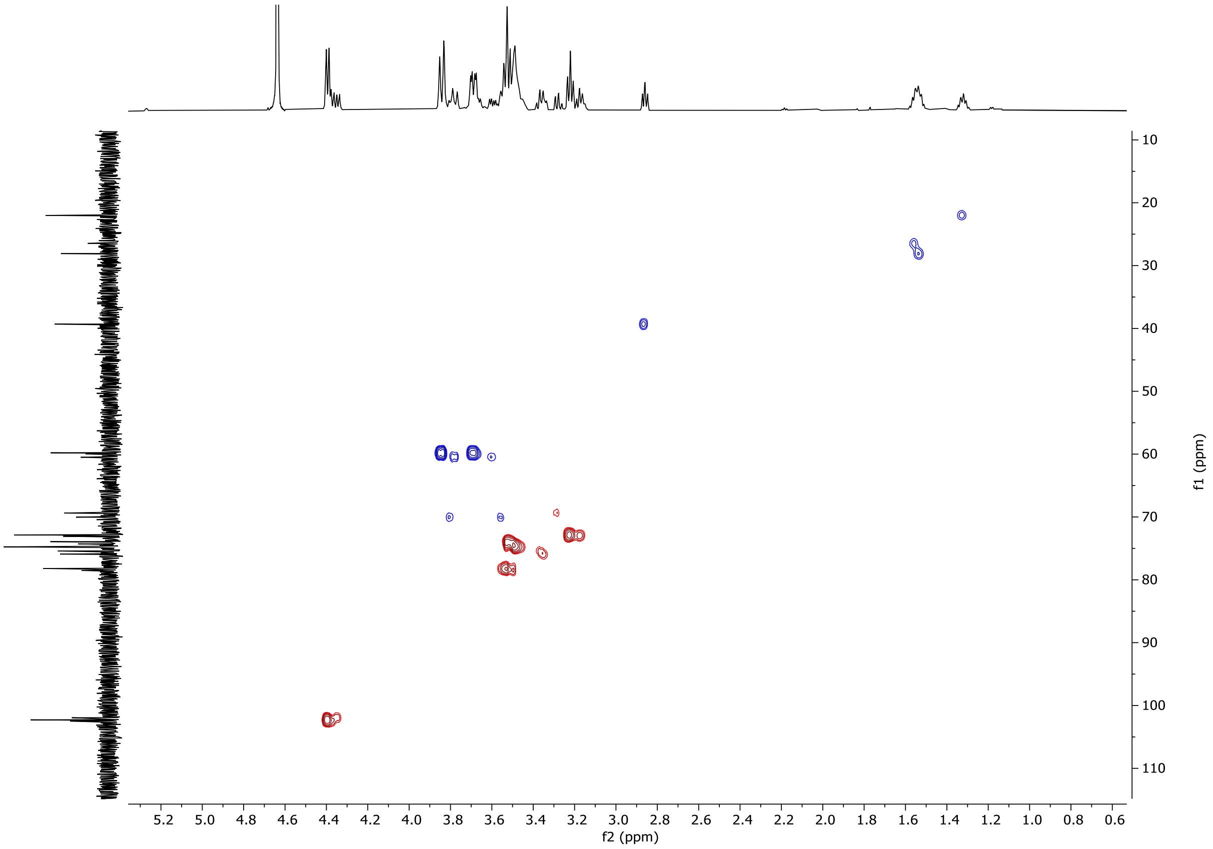

3.6 Synthesis of 6

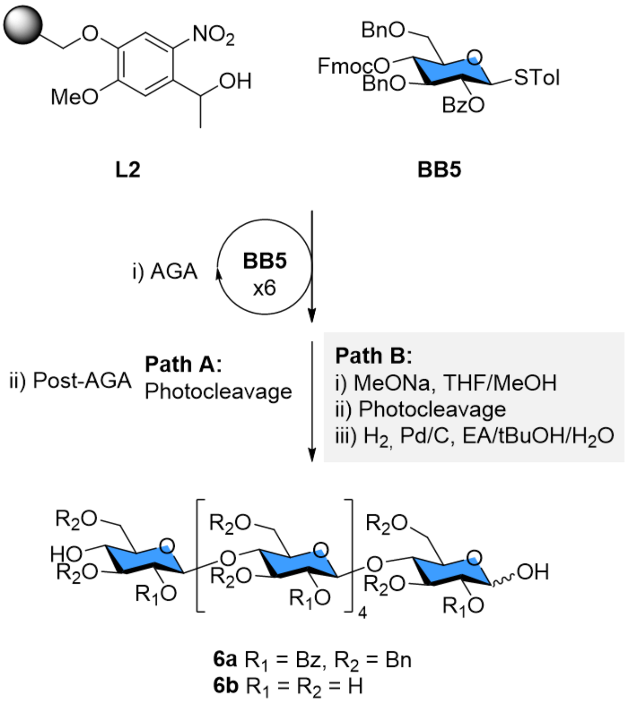

Step	BB	Modules	Notes
AGA	–	A	**L1** swelling
	(**BB5**)x6	(**B**, **C**, **D**, **E1**)x6	**C**: (**BB5**, −20 °C for 5 min, 0 °C for 20 min)
Post-AGA (6a)	–	**G1**, **I**	**I**: (method A2, t_R = 35.4 min)
Post-AGA (6b)		**F**, **G1**, **H**, **I**	**I**: (method C)

Analytical data for compound **6a**: ^{1}H NMR (400 MHz, $CDCl_3$) δ 7.93–7.78 (m, 14H), 7.65–7.56 (m, 6H), 7.56–7.38 (m, 12H), 7.38–7.31 (m, 5H), 7.27 (d, *J* = 4.8 Hz, 4H), 7.20–7.02 (m, 38H), 7.01–6.90 (m, 11H), 5.42 (d, *J* = 3.8 Hz, 0.6H, α-H1), 5.27–5.01 (m, 4H), 5.00–4.84 (m, 6H), 4.77–4.66 (m, 3H), 4.66–4.49 (m, 8H), 4.46–4.34 (m, *J* = 14.7, 2.3 Hz, 5H), 4.30 (d, *J* = 8.3 Hz, 1H), 4.26 (d, *J* = 8.2 Hz, 1H), 4.19 (d, *J* = 8.1 Hz, 1H), 4.17–3.90 (m, 6H), 3.87–3.77 (m, 2H), 3.73–3.59 (m, 2H), 3.58–3.47 (m, 3H), 3.46–3.20 (m, 12H), 3.15–3.10 (m, 1H), 2.93–2.86 (m, 1H), 2.83–2.74 (m, 3H); ^{13}C NMR (101 MHz, $CDCl_3$) δ 167.1, 165.8, 165.0, 164.9, 139.0, 138.8, 138.7, 138.6, 138.2, 138.1, 137.7, 137.6, 137.5, 137.4, 133.3, 133.2, 129.9, 129.8, 129.7, 129.6, 129.3, 128.7, 128.5, 128.4, 128.3, 128.2, 128.1, 128.0, 127.9, 127.8, 127.7, 127.6, 127.5, 127.2, 127.0, 100.3, 100.0, 99.8, 90.3, 81.8, 80.1, 79.9, 77.4, 77.3, 77.1, 76.8, 76.1, 76.0, 75.8, 75.0, 74.6, 74.4, 74.3, 74.1, 73.8, 73.6, 73.5, 73.3, 73.2, 71.1, 69.7, 67.2, 67.1, 53.5, 50.9, 44.6. *m/z* (HRMS+) 2718.039 [M + Na]$^+$ ($C_{162}H_{158}NaO_{37}$ requires 2718.038).

Analytical data for compound **6b**: ^{1}H NMR (400 MHz, D_2O) δ 5.16 (d, *J* = 3.8 Hz, 0.4H, α-H1), 4.60 (d, *J* = 8.0 Hz, 0.6H, β-H1), 4.51–4.41 (m, 5H), 3.96–3.81 (m, 6H), 3.80–3.71 (m, 6H), 3.70–3.48 (m, 16H), 3.47–3.38 (m, 2H), 3.37–3.32 (m, 1H), 3.31–3.19 (m, 5H); ^{13}C NMR (151 MHz, D_2O) δ 102.5, 102.3, 95.7 (β-C1), 91.8 (α-C1), 78.6, 78.4, 78.3, 78.2, 75.9, 75.4, 74.8, 74.2, 74.0, 73.9, 73.8, 73.1, 72.9, 71.2, 71.1, 70.0,

69.4, 60.5, 60.0, 59.8, 59.7; *m*/*z* (HRMS+) 1013.316 [M + Na]$^+$ ($C_{36}H_{62}NaO_{31}$ requires 1013.317). Analytical data for **6b** were in good agreement with those previously reported.[4]

The results obtained for the different syntheses are reported in Figure S9.

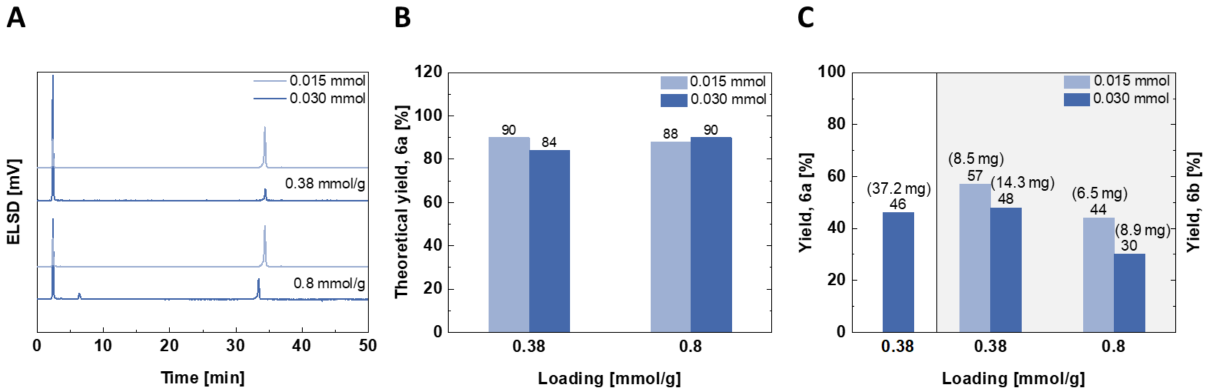

Figure S9 A NP-HPLC chromatograms of the crude compound **6a** after AGA performed on **L2** (ELSD trace, method A1, t_R = 34.6 min). **B** Theoretical yields estimated based on the increased in resin weight after AGA. **C** Isolated yields after post AGA for **6a** (path A) and **6b** (path B).

^{1}H NMR of 6a (400 MHz, CDCl$_3$)

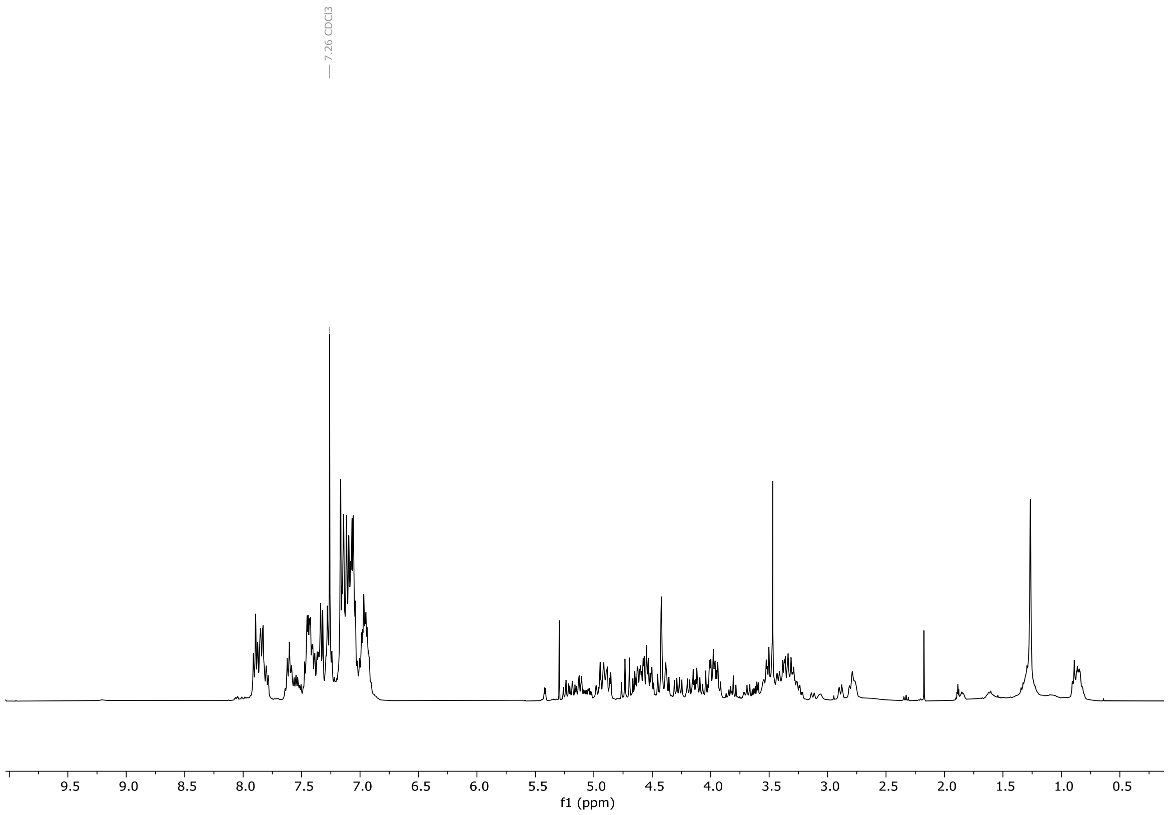

The peaks at 1.27 ppm and 0.93–0.79 ppm belong to residual grease.

^{13}C NMR of 6a (101 MHz, $CDCl_3$)

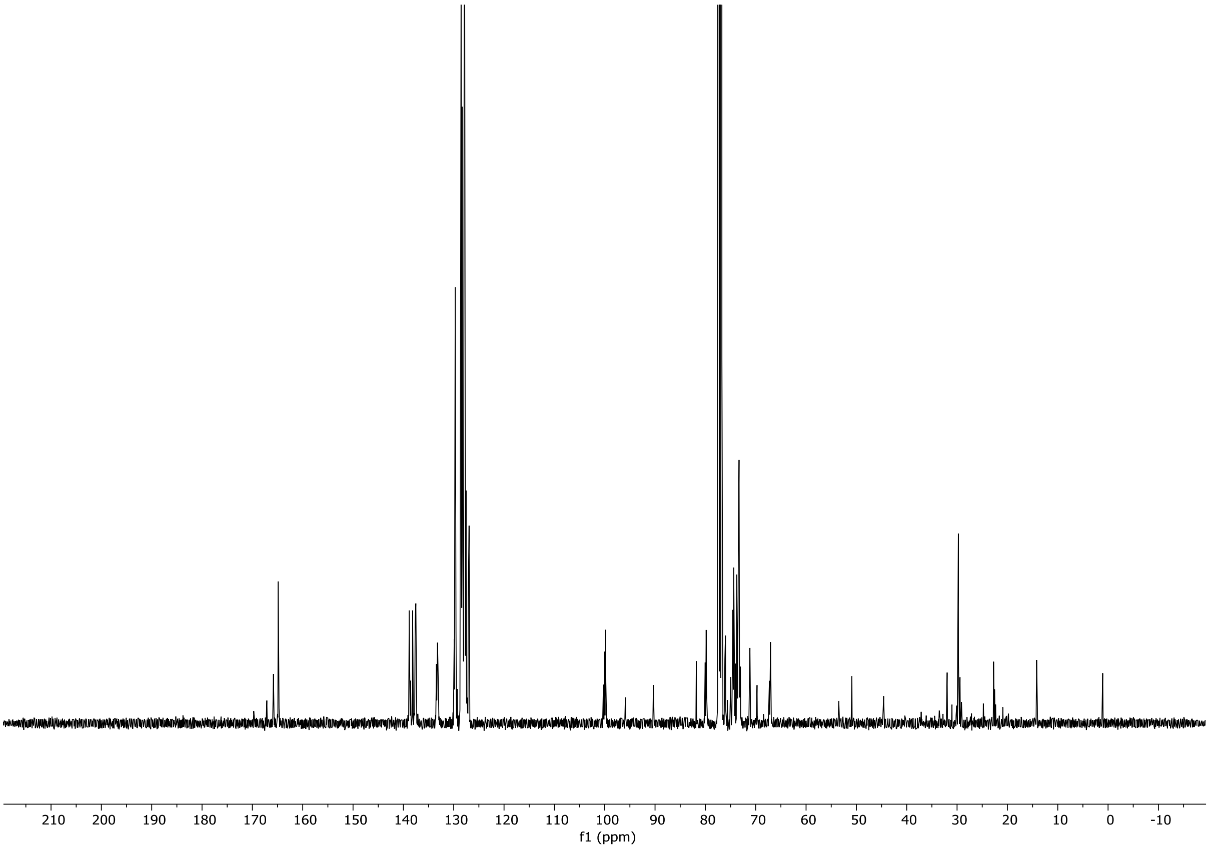

COSY NMR of 6a ($CDCl_3$)

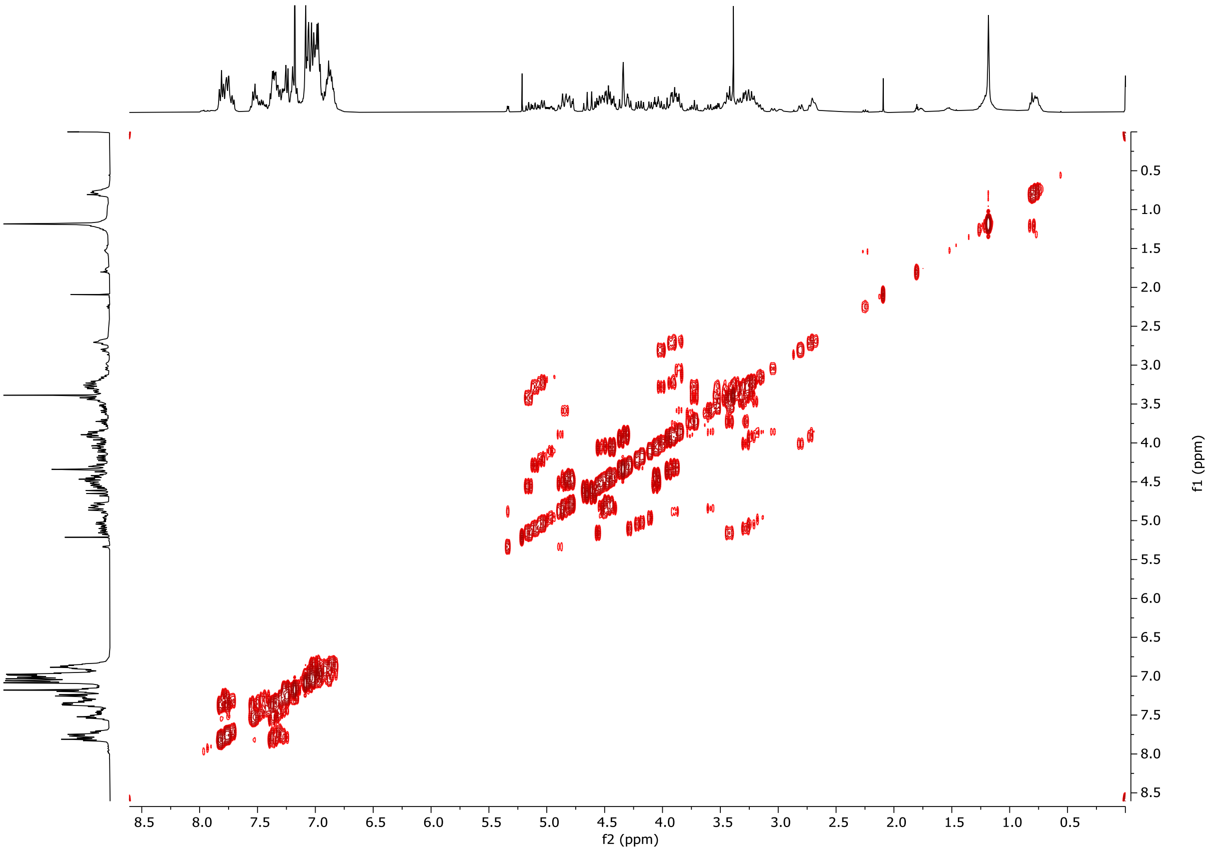

HSQC NMR of 6a ($CDCl_3$)

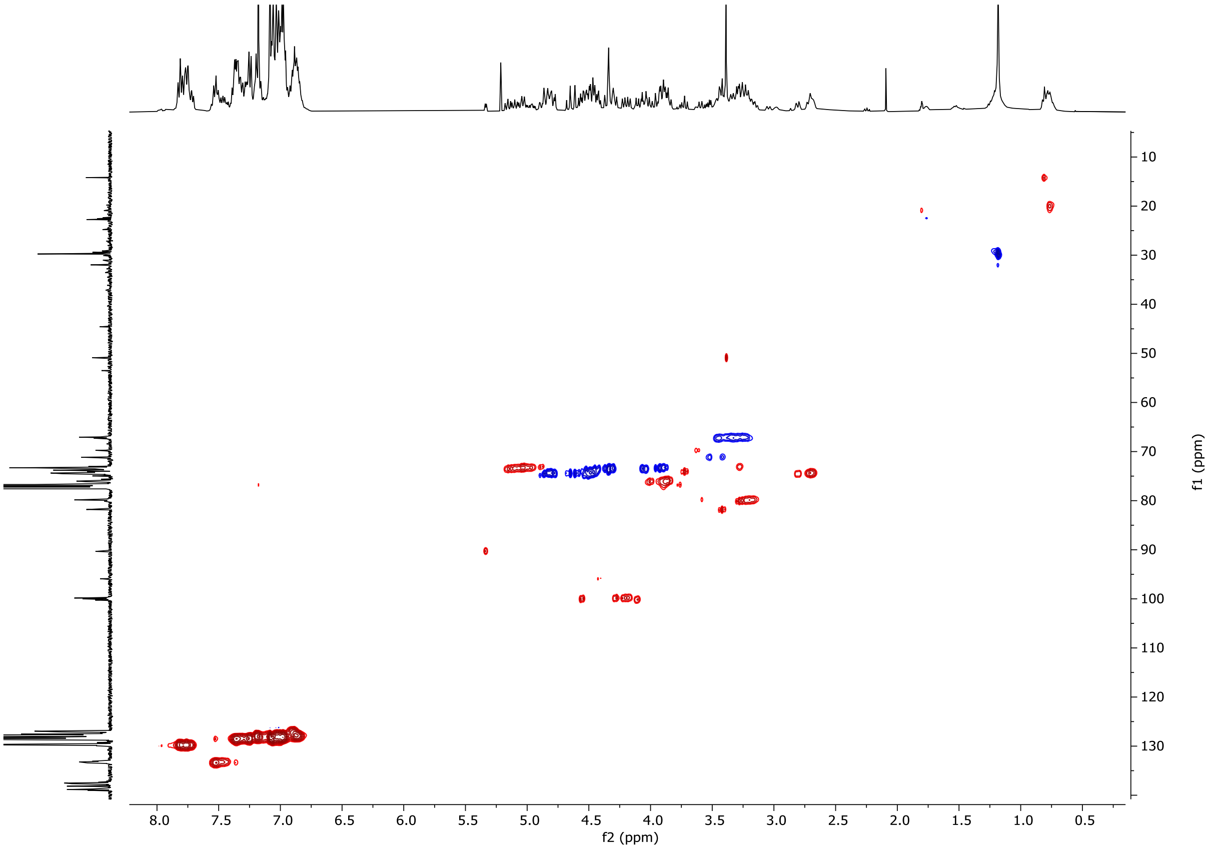

^{1}H NMR of 6b (400 MHz, D_2O)

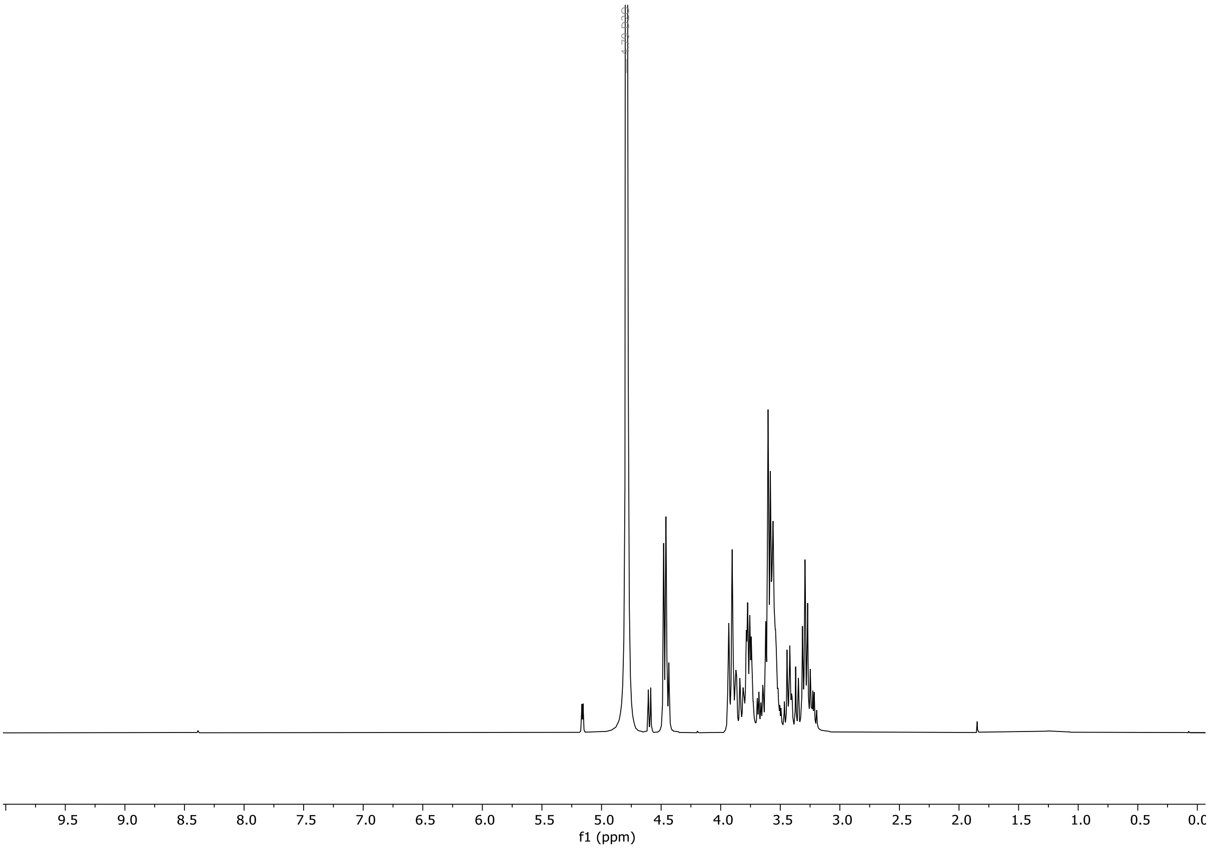

^{13}C NMR of 6b (101 MHz, CDCl$_3$)

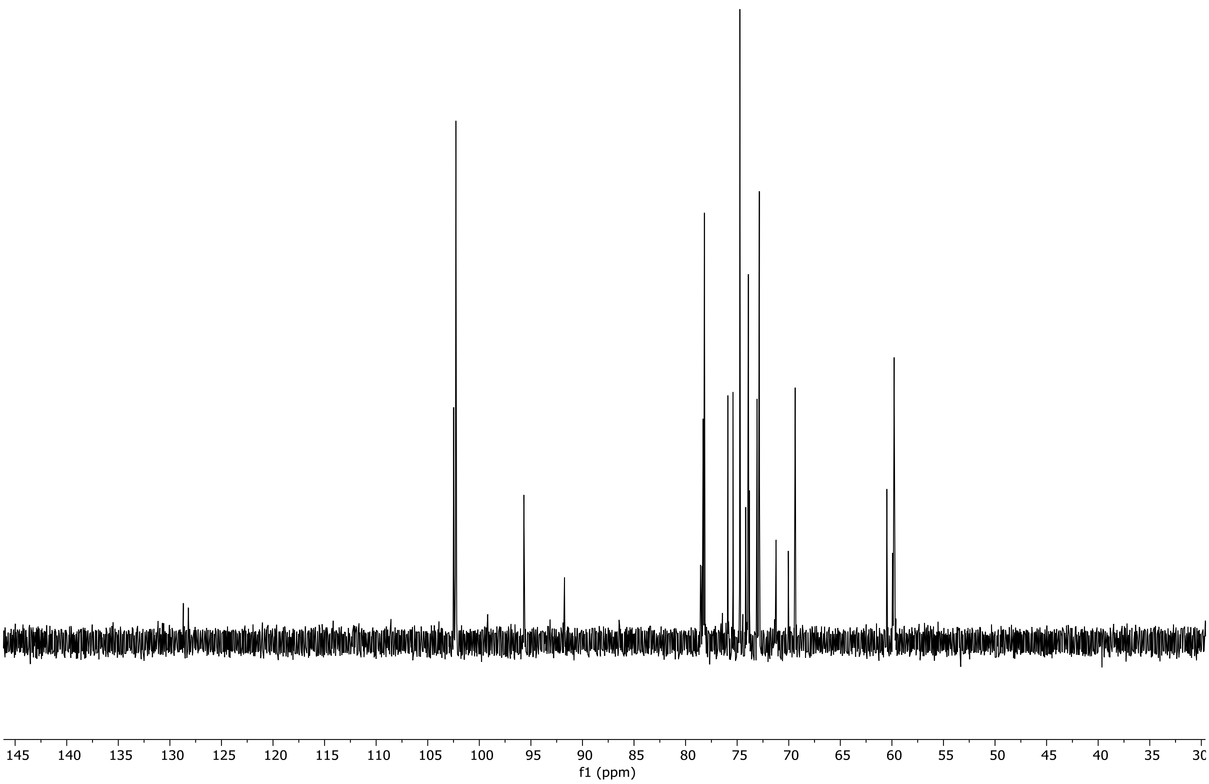

COSY NMR of 6b (CDCl$_3$)

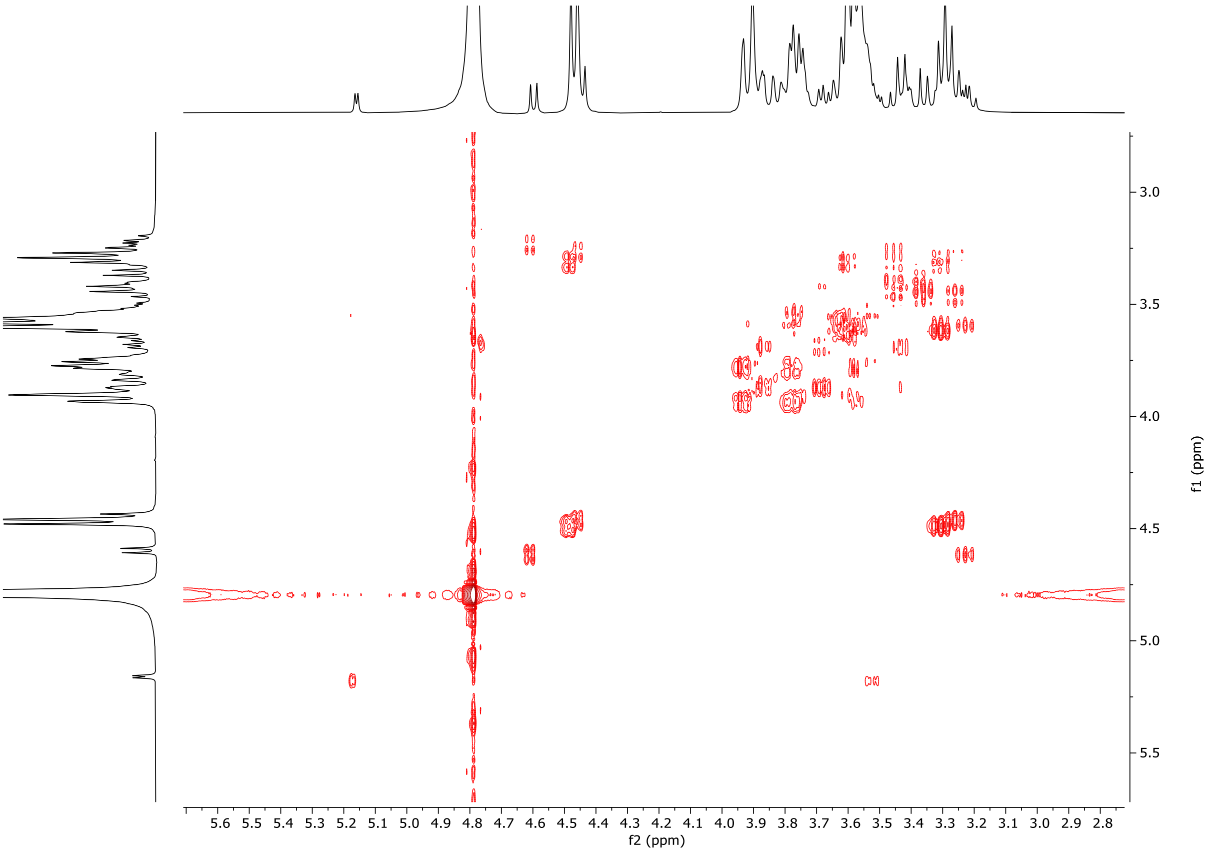

HSQC NMR of 6b ($CDCl_3$)

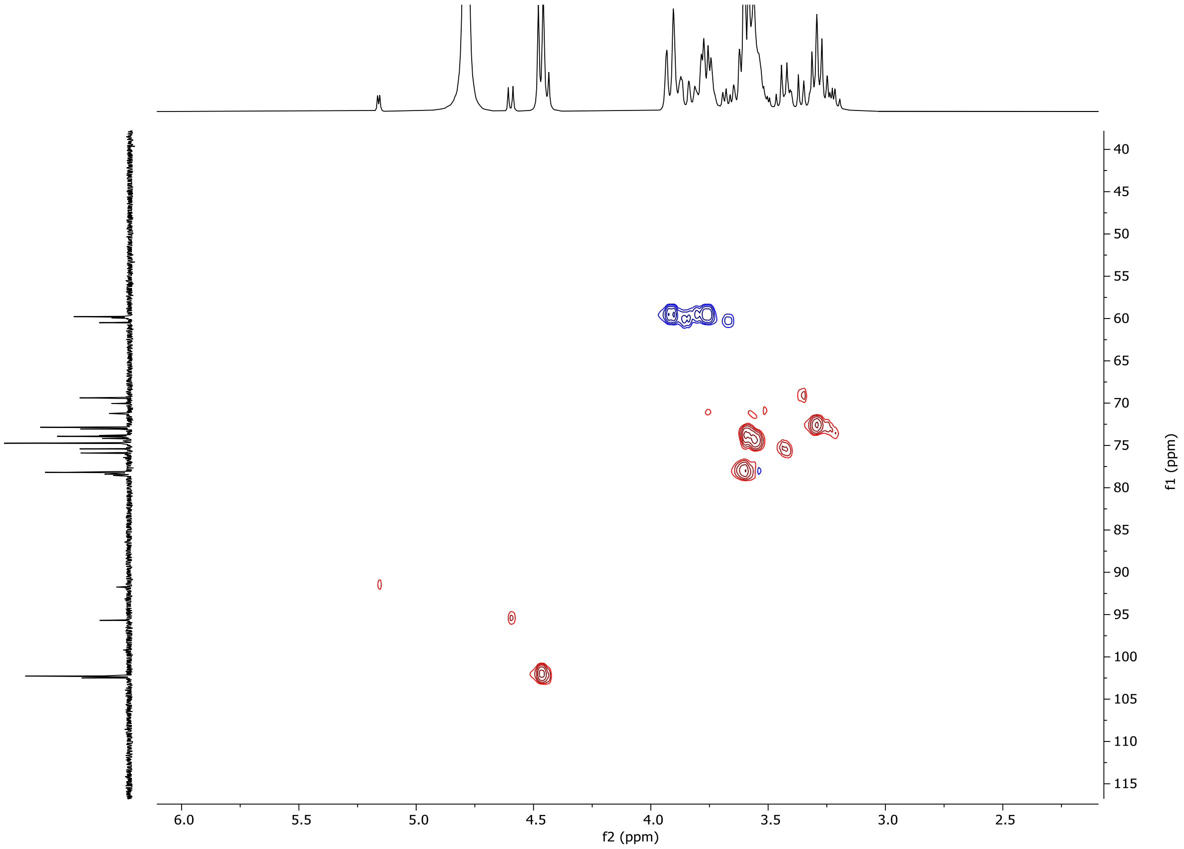

4 References

1. S. Eller, M. Collot, J. Yin, H. S. Hahm, P. H. Seeberger, *Angew. Chem. Int. Ed.* **2013**, *52*, 5858–5861.

2. G. Fittolani, S. Djalali, M. A. Chaube, T. Tyrikos-Ergas, M. C. S. Dal Colle, A. Grafmüller, P. H. Seeberger, M. Delbianco, *Org. Biomol. Chem.* **2022**, *20*, 8228–8235.

3. M. Gude, J. Ryf, P. D. White, *Lett. Pept. Sci.* **2002**, *9*, 203–206.

4. Y. Yu, T. Tyrikos-Ergas, Y. Zhu, G. Fittolani, V. Bordoni, A. Singhal, R. J. Fair, A. Grafmüller, P. H. Seeberger, M. Delbianco, *Angew. Chem. Int. Ed. Engl.* **2019**, *58*, 13127.

5. M. Guberman, M. Bräutigam, P. H. Seeberger, *Chem. Sci.* **2019**, *10*, 5634–5640.

6. M. Hurevich, J. Kandasamy, B. M. Ponnappa, M. Collot, D. Kopetzki, D. T. McQuade, P. H. Seeberger*, Org. Lett.* **2014**, *16*, 1794–1797.

7. K. Ohara, C. C. Lin, P. J. Yang, W. T. Hung, W. B. Yang, T. J. Rachel Cheng, J. M. Fang, C. H. Wong *J. Org. Chem.* **2013**, *78*, 6390–6411.

8. K. Le Mai Hoang, A. Pardo-Vargas, Y. Zhu, Y. Yu, M. Loria, M. Delbianco, P. H. Seeberger, *J. Am. Chem. Soc.* **2019**, *141*, 9079–9086.

9. O. Calin, S. Eller, P. H. Seeberger, *Angew. Chem. Int. Ed.* **2013**, *52*, 5862–5865.

10. M. Delbianco, A. Kononov, A. Poveda, Y. Yu, T. Diercks, J. Jiménez-Barbero, P. H. Seeberger, *J. Am. Chem. Soc.* **2018**, *140*, 5421–5426.
